# Genetic association of beta-lactams-induced hypersensitivity reactions: A systematic review of genome-wide evidence and meta-analysis of candidate genes

**DOI:** 10.1016/j.waojou.2023.100816

**Published:** 2023-09-22

**Authors:** Lalita Lumkul, Pakpoom Wongyikul, Prapasri Kulalert, Mongkhon Sompornrattanaphan, Mongkol Lao-Araya, Mati Chuamanochan, Surapon Nochaiwong, Phichayut Phinyo

**Affiliations:** a Center for Clinical Epidemiology and Clinical Statistics, Faculty of Medicine, Chiang Mai University, Chiang Mai, 50200, Thailand; b Center of Multidisciplinary Technology for Advanced Medicine (CMUTEAM), Faculty of Medicine, Chiang Mai University, Chiang Mai, 50200, Thailand; c Department of Clinical Epidemiology, Faculty of Medicine, Thammasat University, Pathum Thani, 12120, Thailand; d Division of Allergy and Immunology, Department of Pediatrics, Faculty of Medicine, Thammasat University, Pathum Thani, 12120, Thailand; e Division of Allergy and Clinical Immunology, Department of Medicine, Faculty of Medicine Siriraj Hospital, Mahidol University, Bangkok, 10700, Thailand; f Department of Pediatrics, Faculty of Medicine, Chiang Mai University, Chiang Mai, 50200, Thailand; g Pharmacoepidemiology and Statistics Research Center (PESRC), Chiang Mai University, Chiang Mai, 50200, Thailand; h Division of Dermatology, Department of Internal Medicine, Faculty of Medicine, Chiang Mai University, Chiang Mai, 50200, Thailand; i Department of Pharmaceutical Care, Faculty of Pharmacy, Chiang Mai University, Chiang Mai, 50200, Thailand; j Department of Family Medicine, Faculty of Medicine, Chiang Mai University, Chiang Mai, 50200, Thailand; k Musculoskeletal Science and Translational Research (MSTR), Chiang Mai University, Chiang Mai 50200, Thailand

**Keywords:** Beta lactams hypersensitivity, Genetic polymorphism, Genome-wide association study, Systematic review, Meta-analysis

## Abstract

**Importance:**

Beta-lactams (BLs) are the most prescribed antibiotics, being the most frequent cause of drug allergy. However, the association between BL allergy and genetic variations is still unclear.

**Objective:**

This systematic review and meta-analysis aimed to summarize the genetic effects of BL-induced hypersensitivity using existing evidence.

**Methods:**

We searched PubMed, Medline, Scopus, EMBASE, CINAHL, and Cochrane Library from inception to September 15, 2022 with no language restriction. Genetic association studies investigating genetic variant/polymorphism and risk of drug-induced hypersensitivity reactions among individuals receiving BL-antibiotics were included. We excluded studies of acute interstitial nephritis, drug-induced liver injury, serum sickness, and isolated drug fever. Data were comprehensively synthesized and quality of study were assessed using STrengthening the Reporting of Genetic Association Studies (STREGA). The record screening, extraction and quality assessment were performed by two reviewers and discussions were made to resolve discrepancies. The effects of each variant were pooled and evaluated by modified Venice criteria.

**Results:**

A total of 9276 records were identified, and 31 studies were eligible for inclusion. Twenty-seven were candidate-gene association studies (5416 cases and 5939 controls), while the others were next-generation sequencing (NGS) or genome-wide association studies (GWASs) (119 838 cases and 1 487 111 controls). Forty-nine polymorphisms were identified and most of them located in allergic reaction pathways. Meta-analyses of 15 candidate variants in a mixture of both immediate and non-immediate reactions revealed weak genetic effects of rs1801275 (8 studies; n = 1,560; odd ratio 0.73; 95%CI: 0.57–0.93) and rs20541 (4 studies; n = 1,482; odd ratio 1.34; 95%CI: 1.07–1.68) in *IL4R* and *IL13*, respectively. Results from GWASs and NGS identified, and confirmed associations in HLA regions including *HLA-DRA, HLA-B, HLA-DQA, HLA-DRB1,* and *HLA-DRB3*.

**Conclusion:**

Our study summarized genetic evidence influencing BL-induced hypersensitivity and estimated effects of potential variants. We postulated that the genomic studies provide better insights to the mechanism of reactions and suggest potential effects of HLA Class II variants. However, results were inconsistent and unable to generalize in different settings. Further high-throughput studies with a well-defined function, epigenetic interaction, incorporated with clinical factors, would be beneficial for risk identification in BL-induced hypersensitivity.

## Introduction

Beta-lactam (BL)-induced hypersensitivity reaction is a problematic adverse drug reaction (ADR) worldwide due to its high demand in antibiotic prescription. Its prevalence ranged from 7.92% to 14.5%,^1^^,^^2^ in which penicillin and cephalosporin were the most frequent culprits.^3^^,^^4^ Allergy refers to immune-mediated hypersensitivity reactions that are commonly classified into 2 types – immediate and non-immediate responses according to the time of onset. Immediate responses occur within hours after exposure, while non-immediate responses occur after 6 h of exposure.^5^ Indeed, primary hypersensitivity mechanisms are involved in the human leukocyte antigen (HLA) system or immunoglobulin E (IgE)-mediated reactions which can be demonstrated by positive diagnostic tests (i.e., skin test, specific IgE test, or drug provocation test).^6^^,^^7^ Patients with BL-induced hypersensitivity can develop phenotypic symptoms, ranging from urticaria, angioedema, anaphylaxis in immediate reaction, or severe cutaneous adverse events (SCARs) in non-immediate reaction, which are life-threatening.^8^ Based on the culprit antibiotics, BL antibiotics are the top-ranging drug categories that are attributed to SCARs and cause 12.4–44.4% of the total.^9^, ^10^, ^11^

Therefore, BL allergy diagnostic procedures have been highly suggested according to the position paper of the European Academy of Allergy and Clinical Immunology (EAACI),^12^ as well as a practical guide statement from the Canadian Society of Allergy and Clinical Immunology (CSACI).^13^ Several clinical risk prediction models of BL-induced hypersensitivity have been proposed using allergy history and self-report questionnaires to stratify different risk groups of BL allergies.^14^^,^^15^ Data from electronic health records of the United States revealed that up to 12.8% of the population labeled themselves as having an allergy. Yet, more than 95% of those were not truly sensitive and showed negative skin test results.^3^^,^^16^ Consequently, misdiagnosis and delabeling persons at low risk of BL allergy remain vital issues.^17^, ^18^, ^19^

To date, the association of drug-induced hypersensitivity and genetic background has been established. Notably, these associations have been significantly observed in Asian populations, particularly in Han Chinese, Thai, and Malaysian individuals who have received allopurinol and carbamazepine. The pooled effect estimates were found in HLA-B∗58:01 (odd ratio (OR), 96.60; 95% confidence interval (CI), 24.49–381.00) and HLA-B∗15:02 (OR, 79.84; CI, 28.45–224.06), respectively.^20^^,^^21^ As a result, there is a recommendation for genetic screening of persons at risk of drug allergy before treatment with allopurinol and carbamazepine in particular populations.^22^ Several genetic association studies for BL-antibiotics have become apparent in recent years, including candidate gene level and genome-wide association studies (GWASs). However, limited data regarding immune-related genes genotyped and their associations were reported. Moreover, single nucleotide variants (SNVs) or single nucleotide polymorphism (SNP) from interleukin 4 (*IL4*), *IL10*, tumor necrosis factor, HLA, and other immune-related genes were the potential risk of BL-induced hypersensitivity which have been reported in both immediate and non-immediate types.^23^ However, there is limited evidence to leverage a conclusion in this circumstance. As such, we performed a systematic review and meta-analysis based on existing evidence with respect to genetic polymorphisms and subsequent risk of BL-induced hypersensitivity in both immediate and non-immediate reactions.

## Materials and methods

The pre-specified protocol has been published and registered at the International Prospective Register of Systematic Reviews (PROSPERO: CRD42022300283).^24^ The ethical consideration was exempted by the Ethical Committee of Faculty of Medicine, Chiang Mai University (EXEMPTION 8794/2022, FAC-MED-2565-08794) and no informed consent was applicable. The study was conducted following the Human Genome Epidemiology Network for the systematic review of genetic association studies guidance^25^ and reported in line with the Preferred Reporting Items for Systematic Review and Meta-Analysis (PRISMA) statement (Supplemental Table S1).^26^

### Systematic searching and eligible criteria

**Fig. 1:**
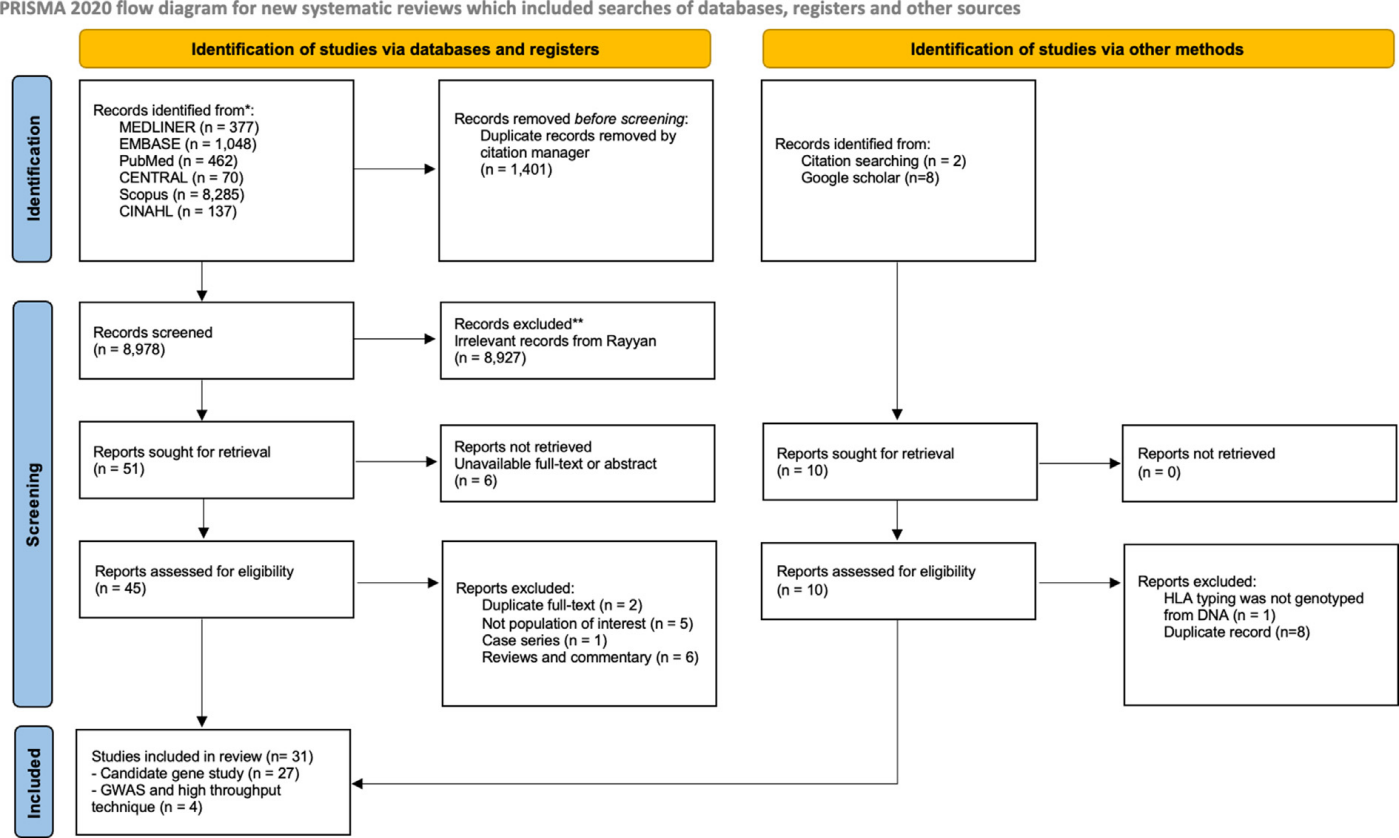
PRISMA flow diagram of systematic review

We searched PubMed, Medline, Scopus, EMBASE, CINAHL, and the Cochrane Library from inception to September 15, 2022 with no language restrictions. Details of the search strategy for each database are provided in Supplementary Table S2. Grey literature, previous systematic reviews, google scholar and reference lists were also browsed for potential eligibility. After deduplicating articles, 2 reviewers (L.L., P.W.) independently screened for potentially eligible articles based on title and abstracts using the Web application—Rayyan.^27^ Then, 2 reviewers (L.L., P.W.) independently appraised the full-text articles against the inclusion/exclusion criteria (Supplementary material and methods). Eligible studies were genetic association studies investigating genetic variant/polymorphism and risk of drug-induced hypersensitivity reactions in both immediate or non-immediate type among individuals receiving BL-antibiotics. Both candidate gene studies and GWASs that provided sufficient information regarding genetic variants, and effect estimates were included in this study. We excluded studies that (i) were case series/case reports, reviews, commentaries, letters to editor; and (ii) investigated the association of genetic markers and other forms of drug-induced hypersensitivity phenotypes (acute interstitial nephritis, drug-induced liver injury, serum sickness, and isolated drug fever). Any disagreement in each process was reached by consultation with the principal investigators (S.N. and P.P.). All records excluded were summarized in the PRISMA flow diagram (Fig. 1).

### Data extraction

Two reviewers (L.L., P.P.) independently extracted relevant information using a predefined data extraction form described in a prespecified protocol.^24^ Study characteristics, sample size, and diagnosis definition were collected, along with the genetic variants and their corresponding loci. Variants were extracted and labeled with the most current name and variant accession number according to National Center for Biotechnology Information (NCBI), and ClinVar databases. We also gathered the number of individuals in each genotype, allele frequencies, and their effect estimates (OR and 95% CI). The final data set was cross-checked and resolved any disagreement by 2 lead reviewers (S.N. and P.P.).

### Outcome of interest

The outcome of interest in this study was BL-induced hypersensitivity based on phenotypes of skin reactions using the EAACI position statement^12^ and the European Network for Drug Allergy (ENDA).^28^^,^^29^ Based on a broad range of symptoms and manifestations, we preferred to use diagnostic tests (ie, skin test, drug provocation test) to assess phenotypes of BL-induced hypersensitivity cases.

### Quality assessment

Two reviewers (L.L., P.P.) independently appraised the quality of each included study using the STrengthening the Reporting of Genetic Association Studies (STREGA).^30^ The STREGA score ranges from 0 to 25, and the high summary score indicates the quality of included studies.^23^

### Data synthesis and meta-analysis

Frequencies of each variant were pooled and checked for Hardy-Weinberg Equilibrium (HWE). Meta-analyses of association and effect size were analyzed in six genetic models^31^ using Stata software version 17.0 (StataCorp, College Station, TX, USA). Moreover, we performed sensitivity analyses which was a leave-one-out method. Plus, predictive intervals and false positive reporting probability (FPRP) of significant variants were also estimated.^32^ Details of statistical analyses were available in supplementary materials.

### Credibility of cumulative evidence

The certainty of cumulative evidence of particular variants in meta-analyses was demonstrated with respect to the modified Venice criteria^33^ and the level of allergy validation test. Evidence credibility was classified as insufficient, weak, moderate, or strong based on the amount of evidence, replications, and biases (supplementary material and methods).

## Results

The systematic search identified 10 379 articles. Of these, 1103 duplicate articles were removed, and 9276 articles remained. By screening titles and abstracts, we identified 45 studies for full-text review. Based on the study selection criteria, 31 studies (27 candidate gene studies,^34^, ^35^, ^36^, ^37^, ^38^, ^39^, ^40^, ^41^, ^42^, ^43^, ^44^, ^45^, ^46^, ^47^, ^48^, ^49^, ^50^, ^51^, ^52^, ^53^, ^54^, ^55^, ^56^, ^57^, ^58^, ^59^, ^60^ 3 GWASs,^61^, ^62^, ^63^ and 1 next-generation sequencing [next-generation sequencing, NGS]-based genotyping^64^) fulfilled the study criteria and were included in this study (Fig. 1).

### Characteristics of eligible studies and quality assessment

Among 27 candidate gene articles, all of them were case-control studies and genotyping was carried out by polymerase chain reaction (PCR)-based methods (supplemental Table S3). The overall individuals included were 11 355 (5416 cases and 5939 controls). Nearly half of the participants were European population (n = 5426), followed by East Asian (n = 5352), African (n = 298), Southeast Asian (n = 117), Western Asian (n = 100), and American (n = 62). In addition, the majority of eligible studies encompassed patients of varying ages, while only 1 study by Singvijarn et al specifically focused on children. Penicillin and cephalosporin are the major culprit drugs among all types of BL-induced hypersensitivity being investigated. Cases were recruited from different etiology in which most of identified cases presented anaphylaxis and urticaria/angioedema, while SCARs were identified in 1 study.^58^ Functional validations to confirm BL hypersensitivity were mainly based on skin tests or serum IgE measurement (21 studies, 77.8%; details are provided in Supplemental Table S4). Control groups were highly heterogenous where more than a half were included based on allergic history and some of them had undergone functional validation.

Table S5 summarized characteristics of eligible high-throughput studies of which 3 studies were GWAS and the other one was NGS-based human leukocyte antigen (HLA) typing. All studies performed replication analyses for their discovered variants in external validation cohorts. These studies employed only European populations which accounted for 31 008 and 88 806 cases in discovery and validation datasets, respectively. One study focused on penicillin allergy while 3 other studies focused on beta-lactams group which include cephalosporins and amoxicillin. Cases were diagnosed based on *in vitro* tests such as skin test, oral provocation, and serum IgE level, except the largest study that recruited participants based on their medical history.

Concerning the quality assessment, evaluation of STREGA score was provided in supplementary materials (Table S6). The score of the included candidate gene studies ranges from 8 to 16 (Table S3), and 17 to 21 in GWAS (Supplemental Table S5).

### Genetic variants that associate with BL-hypersensitivity

Among 27 included candidate gene studies, genetic variants located in 21 genes, contributing to 49 SNVs for both immediate and non-immediate types, and 7 HLA alleles in both of reactions and SCARs (Table 1). The variants were predominantly examined in immune-related genes, including high-affinity IgE receptors, *IL4*, *IL13*, and interferon gamma receptors (*IFNGR*), and IL4 receptor was among the highest interests. Meanwhile, HLA alleles were reported in 2 studies from Chinese^39^ and Thai^58^ populations.

**Table 1:** Variants discovered in candidate gene studies.

Gene	Single nucleotide variant ID	No. of studies (reference)	Culprit Drugs	Hypersensitivity types		
				IR	NIR	SCARs
*FceRIb*	1. rs569108 (E237G): A > G	4^34^^,^^38^^,^^45^^,^^60^	BLs	✓	✓	
	2. rs569108: T > C	2^52^^,^^60^	BLs	✓		
*FceRIG*	1. rs35233990	1^55^	BLs	NA		
	2. rs2070901	1^55^	BLs	NA		
*IL4R*	1. rs1801275 (Gln576Arg, Q576R, Q551R)	8^35^^,^^37^^,^^38^^,^^43^^,^^46^^,^^49^^,^^52^^,^^54^	BLs	✓	✓	
	2. rs1805010 (I50 V, I75 V)	6^37^^,^^38^^,^^41^^,^^43^^,^^49^^,^^56^	BLs	✓	✓	
	3. rs1805015 (S478P)	3^37^^,^^49^^,^^56^	BLs	✓		
*IL4*	1. rs2070874 (−33C/T)	4^41^^,^^49^^,^^57^^,^^60^	BLs	✓		
	2. C-589T	2^38^^,^^44^	BLs	NA		
	3. rs2243291	1^41^	BLs	NA		
	4. rs12186803	1^41^	BLs	NA		
	5. rs10062446	1^41^	BLs	NA		
	6. rs11740584	1^41^	BLs	NA		
*IL13*	1. rs20541 (R130Q): G > A	4^36^, ^37^, ^38^^,^^49^	BLs	✓	✓	
	2. rs20541 (R130Q): C > T	2^52^^,^^56^	BLs	✓		
	3. rs1800925 (−1112C/T)	3^37^^,^^38^^,^^52^	BLs	✓		
	4. rs1881457	1^52^	BLs	✓		
	5.+2044G/A	1^46^	Penicillin	NA		
*IL10*	1. rs1800871 (−819 T > C)	4^38^^,^^40^^,^^41^^,^^52^	BLs	✓		
	2. rs1800872 (−592C > A): C > A	2^38^^,^^52^	BLs	NA		
	3. rs1800872: G > T	1^41^	BLs	NA		
	4. rs1800896 (−1082G/A)	3^38^^,^^40^^,^^52^	BLs	NA		
*IL1R*	1. pst+1970	1^57^	BLs	✓		
*IL21R*	2. −83	1^38^	BLs	NA		
*IL18*	1. −607A/C	1^47^	BLs	✓	✓	
	2. −137G/C	1^47^	BLs	✓	✓	
*IFNGR1*	1. rs11575936 (V14 M)	2^38^^,^^42^	BLs	✓	✓	
*IFNGR2*	1. rs9808753 (Q64R)	2^38^^,^^52^	BLs	NA		
*IFNG*	1. (+874)	1^57^	BLs	✓		
*TNFA*	1. rs1800629	2^49^^,^^59^	BLs	✓		
*NOD1*	1. rs2907749	1^51^	Penicillin, cephalosporin	✓	✓	
*NOD2*	1. rs2066844	1^51^	Penicillin, cephalosporin	✓	✓	
	2. rs2066845	1^51^	Penicillin, cephalosporin	✓	✓	
	3. rs5743293	1^51^	Penicillin, cephalosporin	✓	✓	
*IFIH1*	1. rs1990760	1^48^	Penicillin	NA		
	2. rs3747517	1^48^	Penicillin	NA		
*CYP3A4*	1. rs2242480	1^52^	BLs	✓		
*LACTB*	1. rs2652820 (1523A > G)	2^41^^,^^49^	BLs	✓		
	2. rs1126309	1^41^	BLs	NA		
	3. rs1566660	1^41^	BLs	NA		
	4. rs2729835	1^41^	BLs	NA		
	5. rs34536322	1^41^	BLs	NA		
*STAT6*	1. in 18	1^38^	BLs	NA		
	2. 2SNP3	1^50^	BLs	✓	✓	
	3. rs324011 (6613C > T)	1^52^	BLs	✓		
*LGALS3*	1. rs4644 (P64H)	1^53^	BLs	✓		
	2. rs4652 (T98P)	1^53^	BLs	✓		
	3. rs11125 (Q201H)	1^53^	BLs	✓		
*GC*	1. rs3733359	1^55^	BLs	NA		
*HLA-A* allele		1^58^	BLs	✓	✓	✓
*HLA-B* allele		1^58^	BLs	✓	✓	✓
*HLA-C* allele		1^58^	BLs	✓	✓	✓
*HLA-DRB1* allele		2^39^^,^^58^	BLs	✓	✓	✓
*HLA-DRB3* allele		1^39^	BLs	✓	✓	✓
*HLA-DRB4* allele		1^39^	BLs	✓	✓	✓
*HLA-DRB5* allele		1^39^	BLs	✓	✓	✓

BLs, beta-lactams; GC, Vitamin D binding protein; GWAS, genome-wide association study; IR, immediate reaction; NA, not available; NGS, next generation sequencing; NIR, non-immediate reaction; SCARs, severe cutaneous adverse reactions.

**Fig. 2:**
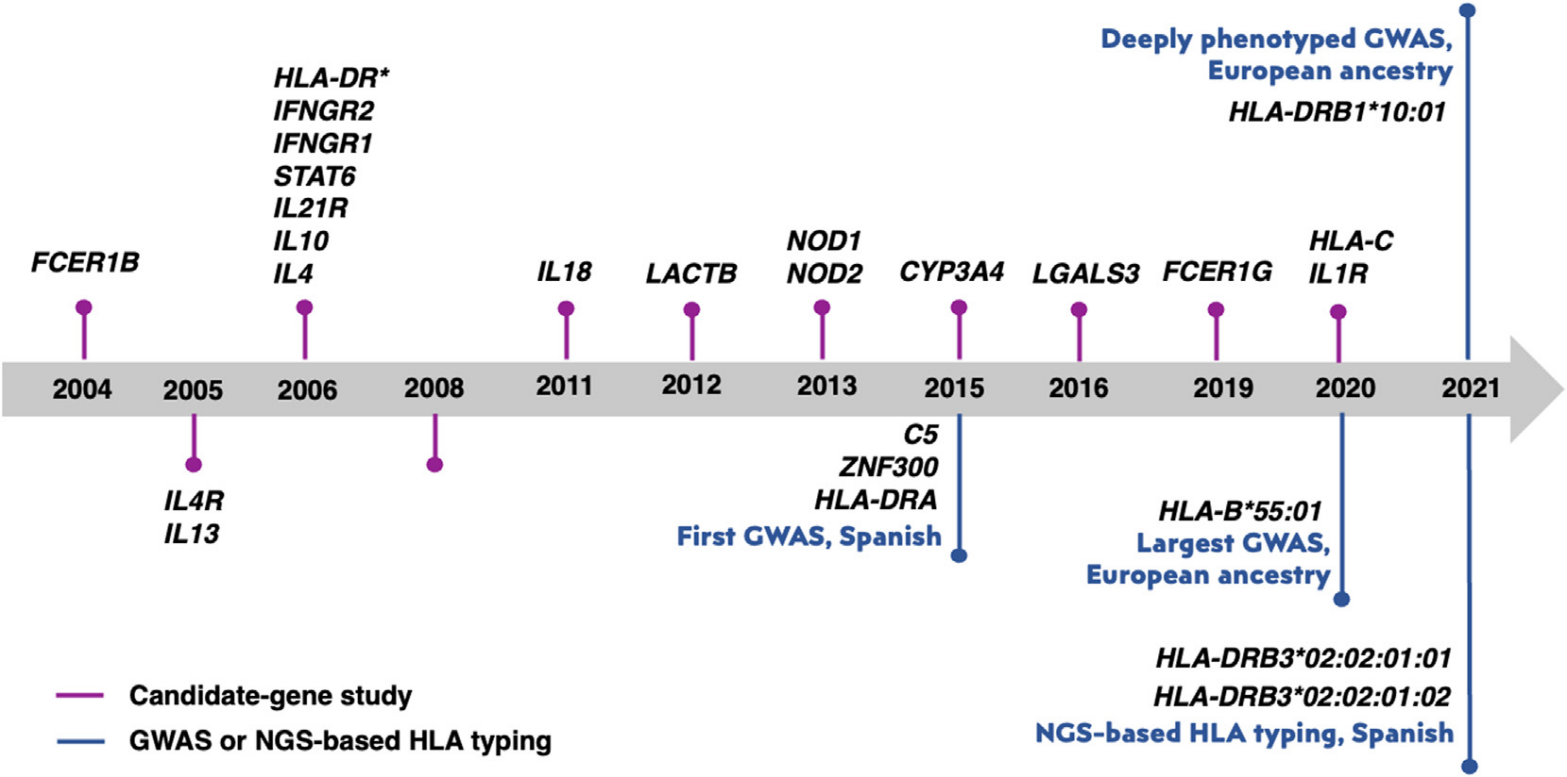
Timeline highlighting first discovery of gene and HLA variants associated with BL-allergy. Upper panel indicated results from candidate-gene study, lower panel showed findings from genome-wide association studies (GWASs) and next- generation sequencing (NGS) based genotyping

On the other hand, in GWAS, more than half of the significant variants were identified in HLA regions (ie, *HLA-DRA:* rs7192, rs8084; *HLA-B∗*55:01*; HLA-DRB1*∗10:01; *HLA-DQA1*∗01:05; *HLA-DRB3*∗02:02:01:02; *HLA-DRB3*∗02:02:01:01) and other cell signaling regulators (ie, *ZNF300* rs4958427*; PTPN22 rs2476601)*. Table 2 summarized the variants that were significantly associated to the disease in external validation analyses. Notably, in the latest study performing by NGS, *HLA*-II variants were significantly associated with non-immediate type hypersensitivity. These highlight the important of *HLA*-II regions in their association with BL-induced hypersensitivity. Also, we demonstrated timeline of genetic variant discovery of BL hypersensitivity reaction in Fig. 2.

**Table 2:** Variants discovered by GWAS and validated in replication cohort.

Genes	SNP ID	Culprit drugs	HR subtypes	Sample sources	Sample size^a^ (case/control)	Allergy functional test	Sequencing technique in validation	Reference
*ZNF300*	rs4958427	BL	IR	Spain, Italy	686/1,486	ST, DPT	Real time PCR	61
*C5*	rs17612							
*HLA-DRA*	rs7192							
	rs8084							
*HLA-B*∗55:01 allele		Penicillins in discover cohort; BLs in validation	NA	European	117,657/1,471,943	Medical history	23andME genotyped by Chip-array	62
*HLA-DRB1*∗10:01 allele		BLs in discovery; Penicillins in validation	IR	European	1028/12,354	ST, DPT	SNP genotyping (TaqMan and Mass array)	63
*HLA-DRB3*∗02:02:01:02 allele		Penicillins	IR,NIR	Spain, Italy	457/1,308	ST, DPT	NGS	64
*HLA-DRB3*∗02:02:01:01 allele								

a Sample size were pooled from discovery and validation cohorts; BLs, beta-lactams; DPT, drug provocation test; HR, hypersensitivity reaction; IR, immediate reaction; NGS, next generation sequencing; NIR, non-immediate reaction; PCR, polymerase chain reaction; SNP, single nucleotide polymorphism; ST, skin test.

### Meta-analysis of genetic effects in candidate gene studies and grading of evidence

From 49 variants, 16 SNVs met our pre-defined criteria for meta-analysis, and a variant in *TNFa* was subsequently excluded due to HWE deviation. Details of analyses in 6 genetic models were provided in Supplemental Table S6. Concerning their estimated p-value and heterogeneity (I^2^), effect estimates of the best genetic model were presented in Table 3, along with allelic model and grading of evidence. Of these, *IL4R* rs1801275 (minor allele (G) in *IL4Ra;* pooled OR, 0.73; 95% CI: 0.57–0.93)^35^^,^^37^^,^^38^^,^^43^^,^^46^^,^^52^^,^^54^ and *IL13* rs20541 (heterozygous (GA vs GG genotype); pooled OR, 1.34; 95% CI, 1.07–1.68)^36^, ^37^, ^38^^,^^65^ demonstrated a significant association with BL-induced hypersensitivity. Nonetheless, given the credibility of these estimated associations, evidence of uncertainty and robustness, our modified Venice criteria revealed weak evidence of their associations to the risk of BL-induced hypersensitivity in both type of reactions) (Table 3).

**Table 3:** Meta-analysis of estimated effects of genetic variants associated with BL-allergy.

Gene	Variant	Allele^a^	Molecular consequence^b^	Number assessed^c^		Allelic contrast model			Best genetic model^d^				Grade of evidence	Modified evidence criteria
				Case	Control	OR (95%CI)	*P*-value	I^2^,% (95%CI)	Model	OR (95%CI)	*P*-value	I^2^,% (95%CI)		
*FceRIb*	rs569108	A > G	Missense variant	253	184	0.90 (0.62,1.32)	0.60	0.0 (0.0,72.89)	Dom	0.29 (0.07,1.19)	0.09	0.0 (0.0,72.89)	BAC	weak
*IL4R*	rs1801275	A > G	Missense variant	812	748	**0.73 (0.57,0.93)**	**0.01**	**32.9 (0.0,70.89)**	**Rec**	**0.70 (0.50,0.99)**	**0.04**	**49.48 (0.00,33)**	ABC	weak
	rs1805010	A > G	Missense variant	1058	1007	1.08 (0.76,1.54)	0.67	85.3 (66.38,91.45)	Dom	1.15 (0.77,1.72)	0.51	66.0 (0.0,83.79)	ACB	weak
	rs1805015	T > C	Missense variant	749	704	0.99 (0.75,1.32)	0.97	40.5 (0.0,82.46)	Dom	1.25 (0.62,2.54)	0.53	0.0 (0.0,72.89)	ABC	weak
*IL4*	rs2070874	C > T	5 prime UTR	381	456	0.73 (0.21,2.61)	0.63	67.4 (NA)	Dom	0.52 (0.19,1.45)	0.21	0.0 (NA)	BBC	Insufficient
	C-589 T	C > T	NA	94	99	1.55 (0.92,2.61)	0.10	0.0 (NA)	Dom	1.93 (0.90,4.15)	0.09	0.0 (NA)	BAC	Insufficient
*IL13*	rs20541	G > A	Missense variant	748	734	1.17 (0.98,1.40)	0.09	0.0 (0.0,67.91)	Het	**1.34 (1.07,1.68)**	**0.01**	**0.44 (0.0,68.02)**	AAC	weak
	rs1800925	C > T	5 prime UTR	258	331	1.05 (0.59,1.86)	0.88	63.9 (0.0,87.64)	Dom	0.43 (0.10,1.82)	0.25	29.8 (0.0,80.13)	BBC	weak
*IL10*	rs1800871	T > C	2 KB upstream variant	210	160	0.95 (0.69,1.32)	0.76	0.0 (0.0,72.89)	Dom	0.78 (0.41,1.50)	0.46	0.0 (0.0,72.89)	BAC	weak
	rs1800872	C > A	2 KB upstream variant	108	74	1.05 (0.67,1.65)	0.83	0.0 (NA)	Co-do	0.81 (0.43,1.51)	0.50	0.0 (NA)	BAC	Insufficient
	rs1800896	A > G	2 KB upstream variant	210	160	1.08 (0.55,2.11)	0.83	65.9 (0.0,88.10)	Homo	1.18 (0.30,4.61)	0.81	61.8 (0.0,87.16)	BBC	weak
*IFNGR1*	rs11575936	G > A	Missense variant	188	132	0.33 (0.06,2.39)	0.27	53.6 (NA)	Het	0.29 (0.06,1.38)	0.12	32.6 (NA)	BBC	Insufficient
*IFNGR2*	rs9808753	A > G	Missense variant	108	74	0.98 (0.60,1.59)	0.92	0.0 (NA)	Co-do	1.33 (0.70,2.52)	0.38	0.0 (NA)	BAC	Insufficient
*TNFA*	rs1800629	G > A	2 KB upstream variant	509	600	1.07 (0.85,1.34)	0.59	0.0 (NA)	Dom	1.13 (0.84,1.52)	0.42	0.0 (NA)	AAC	Insufficient
*LACTB*	rs2652820	G > A	Intron variant	363	379	0.75 (0.43,1.31)	0.32	70.7 (NA)	Rec	0.71 (0.36,1.42)	0.34	76.5 (NA)	BBC	Insufficient

a Reference alleles > minor alleles.

b Molecular consequence reported based on NCBI: dbSNP database.

c Studies that control population significantly deviated from Hardy-Weinberg Equilibrium were excluded.

d Best model defined by its *P*-value and I^2^; OR = Odds ratio; CI = confidence interval; A = Adenine; T = Thymine; C=Cytosine; G = Guanine; UTR = untranslated region; Dom = Dominant; Rec = Recessive; Het = Heterozygous; Homo = Homozygous, Co-do = Co-dominant; NA = not applicable; Bold values represented significant association.

Further subgroup analyses regarding to ethnicity (ie, Asian and non-Asian) were performed and no association was observed in *IL4R* rs1801275. However, the association remains significant in non-Asian population of *IL13*: rs20541, G > A (Supplemental Table S7-8). Moreover, the latter variant demonstrated acceptable false positive reporting probability which concludes noteworthiness of the effect estimated (Supplemental Table S7-8). The results were consistent in sensitivity analyses using a leave-one-out approach (Supplemental Table S9-10).

## Discussion

A growing body of evidence has found that genetic background has influenced the risk of BL-induced hypersensitivity, especially in genes regulating allergic reactions; however, their genetic effects remain inconclusive.^66^ This present systematic review and meta-analysis of genetic association studies illustrated the effects of genetic predisposition in BL-induced hypersensitivity. Meta-analysis revealed significant association of the reactions and *IL4R* and *IL13* polymorphisms, but the resulting effect sizes were small and highly heterogenous, providing weak evidence of effect estimated. In addition, given that none of these identified variants were replicated in subsequence GWASs, drawing conclusion of these genetic effects of BL hypersensitivity may render several concerns.

In comparison to previous reviews,^23^^,^^66^ our study provided an expanded analysis of the pooled effects for 15 SNVs associated with BL-induced hypersensitivity. Because most of eligible studies recruited a mixture of immediate and non-immediate hypersensitivity population and did not provide data separately, our analyses pooled the results from both reactions. As a result, we found that 2 variants in *IL4R* (rs1801275; protective factor) and *IL13* (rs20541; risk factor) were significantly associated with BL-induced hypersensitivity (mixed reactions). Since *IL4R* and *IL13* play major roles in IgE-mediated activation for allergy, they have been studied widely in different populations since 2004 and have become a promising therapeutic target for allergic reactions in recent years.^67^ In particular, rs1801275 polymorphism has been reported as a risk factor for allergic rhinitis^68^ and cancer susceptibility,^69^ where some other evidence found controversial results.^70^^,^^71^ In this study, we revealed a protective effect of rs1801275 polymorphism in decreasing the risk of BL-induced hypersensitivity. However, this finding seems to be susceptible to ethnicity and evidence of uncertainty; therefore, the effect of association was lost in subgroup analysis.

Interestingly, for rs20541, we found that this polymorphism increases the risk of BL-induced hypersensitivity. Like other studies, this polymorphism demonstrated promising genetic effect to various allergic diseases including asthma,^72^ allergic rhinitis,^68^ atopic dermatitis, and even with alternation of total serum IgE levels.^73^ According to dbSNP, this risk allele A accounts for 0.21 of total allele frequency in global population (from 367 982 sample size). Still, despite its promising results in allergic diseases and high frequency in global population, these variants remain unidentified in high throughput studies.

Since the results were pooled from hypothesis-based genetic studies where their proposed variants were restrained to IgE/IL4-IL13 pathways, other hidden variants with hypothesis-free polymorphisms remained undiscovered. As the advancement of Omic technology, high-throughput genome sequencing has revolutionized the genetic study approach into GWAS. Instead of selecting a few variants to study, GWAS is a hypothesis-free method that the whole SNPs in genome, even in non-coding region, are compared. This has been acknowledged among statistical geneticists that GWAS makes findings from candidate gene approach outdate.^74^

Thus, attention has brought to genome study in BL-allergy during the past seven years. However, this study approach requires massively large sample size.^75^ The first published GWAS of BL-allergy with relatively small participants and inadequate statistical power, thus provided a marginal significance of associations of HLA regions.^61^ Later, with tremendously large study cohorts, Krebs et al and Nicoletti et al revealed promising associations in which polymorphisms of *HLA-B*∗55:01 and *HLA-DRB1*∗10:01 were addressed. The regions were typically associated with hypersensitivity of several drugs such as carbamazepine,^21^ allopurinol^20^ and flucloxacillin.^76^ In addition to that, hepatotoxicity induced by flucloxacillin, an antibiotic in beta-lactams, also associated with *HLA-B*∗57:01 which is considered to be class I restricted CD8 T cell-mediated reaction. In terms of hypersensitivity reaction, BL involves a wide range of immune response in antigen presentation, recognition and IgE production.^23^ Furthermore, HLA class II (*HLA-DP*, *HLA-DQ*, *HLA-DR*) plays important roles in activation of T-helper 2 cell from which IL4, IL5, IL13 were secreted resulting in IgE production B-cell.^77^ This homology of HLA/IgE activation was also observed in asparaginase (*HLA-DRB1*∗07:01) hypersensitivity.

Regarding the effect size of these GWASs, even with recent NGS-based HLA typing, the SNPs identified were inconsistent. Though the SNPs located in the HLA region can functionally relate to allergic reactions, the substantial effect estimates of these variants were relatively small. It is important to note that large effect variants are generally rare or non-existent. Otherwise, they would be identified early, like other Mendelian diseases. The results of BL-induced hypersensitivity are consistent with other non-communicable complex diseases, such as cardiovascular disease that require large samples to explore genetic effects.^78^ These also suggested the small effect of genetic markers, and estimating the risk effects using only a single nucleotide variant was insufficient to support their etiology and prediction. However, combined genetic effects plus other epigenetic, environmental, and clinical factors would be able to help identify risk groups.

### Strengths and limitations

Our results provided the pooled estimates of 2 immune-related variants and have incorporated relevant findings from genomic research in understanding BL-induced hypersensitivity. However, it is important to note that this systematic review and meta-analysis contains several limitations. First, owing to a limited number of included studies and clinical data given, we were unable to perform subgroup analyses involving specific genetic variants and each individual BL antibiotic. Furthermore, most of the participants in this study were based on a mixture of immediate and non-immediate type reactions, and only three studies acknowledged differential analysis in delayed hypersensitivity cases. Hypersensitivity reaction was defined based on broad range criteria, and the lack of diagnostic confirmation in control groups; therefore, the effect estimates for particular hypersensitivity subtype could not be established and suggested weak level of evidence. Second, because of different methodologies regarding genotyping, and annotation of HLA variants in candidate gene studies (ie, PCR-SSO and PCR-SSP were not comparable), we cannot comprehensively analyze all the candidate gene polymorphisms in this region. Third, the majority of candidate gene studies were conducted in Chinese populations while GWASs were primarily based on European ancestry. Therefore, this could potentially limit the replicability and generalizability of our findings. Further comprehensive GWAS meta-analysis and studies encompassing diverse populations are imperative to validate our findings.

### Implications for practice

Considering substantial influence of the environment on BL sensitization, genetic study of BL-induced hypersensitivity should take special thought to genetic and clinical or environmental factors interaction. Besides, further key concerns include disease incidence, positive predictive value of genetic test, cost-effectiveness, and clinical utility in randomized controlled trial. Thus, risk stratification to render low-risk patients is favorable, yet challenging.^18^ A rigidly helpful delabeling strategy in labeled patients can help prevent alternative antibiotic consumption, higher costs, and extended length of stay.^4^

### Potential future research

In contrast to allopurinol and carbamazepine, which exhibit strong associations to HLA, the genetic effects of BL were sparse and small.^21^ Thus, single variant study would not be certainly helpful. Modern genomic approach still encounters between-study inconsistencies and many of discovered SNPs were unable to replicate in validation dataset.^79^ The study approach for validating GWAS results proceeded cautiously under valid biological processes, with adequately powered *HLA*-II haplotype association and well-defined population can still be relevant.^80^ Also, pathologic mechanisms are needed to be explored along with multiple risk predictors, epigenetic study in combination with clinical and environmental factors.

## Conclusion

This systematic review and meta-analysis highlight the genetic effects of BL-induced hypersensitivity. Studies conducted using a candidate-gene approach showed cumulative results indicating associations between BL hypersensitivity reaction and *IL4R* (rs1801275) and *IL13* (rs20541) polymorphisms. However, the uncertainty of data and study approach remains an issue. Extrapolating genetic background to clinical use is still challenging. A combination of genetic and clinical risk factors may facilitate the prediction of BL-induced hypersensitivity to stratify risk in patients and potentially enhancing drug allergy management.

## Abbreviations

A, Adenine; ADR, Adverse drug reaction; BL, Beta-lactams; C, Cytosine; CI, Confidence interval; Co-do, Co-dominant model; Dom, Dominant model; FPRP, False positive reporting probability; G, Guanine; GWAS, Genome-wide association study; Het, Heterozygous model; HLA, Human leukocyte antigen; Homo, Homozygous model; HR, Hypersensitivity reaction; HWE, Hardy-Weinberg equilibrium; IgE, Immunoglobulin E; IL, interleukin; IR, Immediate type reaction; NIR, Non-immediate type reaction; OR, Odd ratio; PCR, Polymerase chain reaction; PCR-SSO, PCR-sequence specific oligonucleotide probe; PCR-SSP, PCR-sequence specific primers; Rec, Recessive model; SCAR, Severe cutaneous adverse reaction; SNP, Single nucleotide polymorphism; SNV, Single nucleotide variant; STREGA, STrengthening the Reporting of Genetic Association Studies; T, Thymine; UTR, Untranslated region.

## Funding

This research was partially supported by 10.13039/501100002842Chiang Mai University and 10.13039/501100010731Faculty of Medicine, Chiang Mai University.

## Availability of data and materials

Supplementary information accompanies this paper can be found in Appendices.

## Author contribution

LL performed record screening and extraction, meta-analysis and prepared the manuscript. PW extracted data and assessed the study quality. PK, MS, ML, and MC discussed the quality of evidence and revised the manuscript. SN and PP conceptualized, supervised and revised statistical analyses.

## Authors’ consent for publication

All the authors have approved the manuscript and agree with its submission to the journal.

## Institutional review board statement

The ethical approval was exempted by the Ethical Committee of the Faculty of Medicine, Chiang Mai University (EXEMPTION 8794/2022, FAC-MED-2565-08794) and no informed consent was applicable.

## Declaration of competing interest

M. Sompornrattanaphan has received honoraria for scientific lectures from A. Menarini, Astra-Zeneca, GSK, Takeda, and Viatris, and research supports from Abbott and Sanofi. The company had no role in the design of the study; in the collection, analyses, or interpretation of data; in the writing of the manuscript, or in the decision to publish the results. The others declare no conflict of interest.
