# Supplementary material for: Genetic association of beta-lactams-induced hypersensitivity reactions: A systematic review of genome-wide evidence and meta-analysis of candidate genes
Source: World Allergy Organ J. 2023 Sep 22;16(9):100816. doi: 10.1016/j.waojou.2023.100816 (PMC10541471; doi:10.1016/j.waojou.2023.100816)
Supplement: Multimedia component 1 [file mmc1.doc]

**Supplement materials and methods**

**Systematic Searching of the Literature**

We conducted a systematic search based on the comprehensive search strategy guidelines [1] and the pre-specified search strategy proposed in previous protocol [2]. Briefly, the search was executed in collaboration with experienced librarian using standard biomedical databases including PubMed, Medline, Scopus, EMBASE, CINAHL, and the Cochrane central register of Controlled Trials (CENTRAL) from inception with no language restrictions. Unpublished articles aside from commercial publishers were approached as grey literature. Citations from relevant systematic review, narrative review, and obtained records were screened for potentially eligible studies.

**Selection process and eligible criteria**

Deduplicated records obtained from systematic search were retrieved and screened using a web application for systematic reviews, Rayyan [3] as described in previous protocol [2]. Full-text articles of inclusion were accessed and two reviewers (L.L., P.W.) evaluated their eligibility based on pre-specified criteria: (i) randomized trials and observational studies that were case–control, cross-sectional, or cohort studies; (ii) association studies of genetic markers/polymorphism and the risk of drug-induced hypersensitivity reactions among individuals receiving beta-lactams therapy regardless of age and indications; (iii) sufficient information provided to calculate the association between genetic variants and risk estimate (e.g., odds ratio (OR), allele frequencies). Either candidate gene studies or Genome-wide association studies (GWASs) will be included. Exclusion criteria were studies that (i) were case series/case reports, reviews, commentaries, letters to editor; and (iii) investigated the association of genetic markers and other forms of drug- induced hypersensitivity phenotypes (acute interstitial nephritis, drug-induced liver injury, serum sickness, and isolated drug fever). Studies that met inclusion criteria with sample size less that 100 were included due to low availability of records. Discrepancies were discussed and consensus with methodological and clinical experts. All records excluded were summarized in PRISMA flow diagram (Figure 1).

**Outcomes of interest**

The primary outcome was the determined phenotypes of relevant beta-lactams-induced hypersensitivity reactions. Symptoms such as rash, urticaria, angioedema, anaphylaxis, severe cutaneous adverse reactions (SCARs) including acute generalized exanthematous pustulosis (AGEP), drug rash with eosinophilia and systemic symptoms (DRESS), Stevens–Johnson syndrome (SJS), and toxic epidermal necrolysis (TEN) were included as immediate reaction (IR) or delayed reaction (DR) according to primary definition of eligible records. Comprehensive assessment of immunological testing was evaluated by allergists and dermatologists (P.K., M.S., M.L., and M.C.) for which the ascertainment of hypersensitivity test was rated . Patient diagnosis criteria using medical history/ questionnaires, and validation testing (serum immunoglobulin E (IgE) measurement, skin prick test, intradermal and/or oral provocation) were classified.

**Data extraction**

Data were independently extracted by two authors (L.L., P.P.) following the published protocol [2]. Briefly, study characteristics including authors and year of publication, geographic region and country, study design, study type (i.e., candidate gene study or Genome-wide association study), sample size, study population were derived. Participant population, gender, ethnicity, allergic

history, and case ascertainment definition were provided. Genotyping method associated genetic variants and their corresponding loci were extracted using the most current name and variant accession number according to National Center for Biotechnology Information (NCBI) databases. Number of participants in each genotype were retrieved, affected and non-affected allele frequencies and their effect size (OR and 95% confidence interval) were retrieved. Also, laboratory functional investigations were provided.

**Quality assessment**

Quality of eligible studies were determined independently by two reviewers (L.L., P.P.) following the recommendations framework of STrengthening the Reporting of Genetic Association Studies (STREGA) [4]. The total 25 concerns of strengths and weakness evaluation were reviewed as previously described [5]. Any discrepancies were discussed consensus with methodological expert (S.N.) and STREGA scores for eligible studies were reported.

**Data synthesis and meta-analysis**

Genotype frequencies of each variant were aggregated, and meta-analysis for common effect (Odds ratio, OR) was performed following steps suggested in [6]. All analyses were conducted using Stata software version 17.0 (StataCorp, College Station, TX, USA). Initially, allele frequencies from each study were estimated for Hardy-Weinberg Equilibrium (HWE). Studies that significantly deviated from HWE (P-value <0.05) in control population were excluded from quantitative analysis. Allelic and genotypic effects were estimated based on six genetic models including allele contrast, homozygous, heterozygous, dominant, recessive, and co-dominant [6]. The effects were analyzed using random effect model by DerSimonian Laird method if heterogeneity (I2) was presented (I2 > 25%); otherwise, a fixed effect model by Mantel-Haenszel methods were used. Forest plots were presented, and subgroup analysis was specified by ethnicity as non-Asian, and Asian groups. To determine robustness of results, sensitivity analysis was addressed by cumulative meta-analysis and leave-one-out method. Predictive value, and false positive reporting probability (FPRP) were calculated [7].

**Assessment of cumulative evidence**

Cumulative evidence incorporated in meta-analysis were evaluated for epidemiological credibility using modified Venice criteria [8] together with level of allergy validation test. Estimated effects were rated as insufficient, weak, moderate or strong. If the pooled effects were assessed from less than three studies, credibility was downgraded to insufficient regardless of any protection from bias. Data pooled from three or more studies were graded. Amount of evidence and replication were assessed based on pooled sample size and heterogeneity, the I2, respectively. Protection from bias was evaluated in respect of phenotype definition, genotyping, population stratification and selective following the criteria [8]. Based on EAACI position paper [9], any allergy definition not addressing drug provocation test or severe allergic symptoms was considered unclear phenotype.

**Ethics and dissemination**

This systematic review and meta-analysis comprised of available published data which human subjects were not directly involved. Thus, the ethical exemption was approved by Ethical Committee of the Faculty of Medicine, Chiang Mai University (EXEMPTION 8794/2022, FAC-MED-2565-08794).

**References**

1. Cooper C, Booth A, Varley-Campbell J, Britten N, Garside R. Defining the process to literature searching in systematic reviews: a literature review of guidance and supporting studies. BMC Medical Research Methodology. 2018;18(1):85.

2. Lumkul L, Chuamanochan M, Nochaiwong S, Sompornrattanaphan M, Kulalert P, Lao-Araya M, et al. Genetic Association of Beta-Lactams-Induced Hypersensitivity Reactions: A Protocol for Systematic Review and Meta-Analysis. Genes (Basel). 2022;13(4).

3. Ouzzani M, Hammady H, Fedorowicz Z, Elmagarmid A. Rayyan—a web and mobile app for systematic reviews. Systematic Reviews. 2016;5(1):210.

4. Little J, Higgins JP, Ioannidis JP, Moher D, Gagnon F, von Elm E, et al. STrengthening the REporting of Genetic Association Studies (STREGA)--an extension of the STROBE statement. Genet Epidemiol. 2009;33(7):581-98.

5. Guéant JL, Romano A, Cornejo-Garcia JA, Oussalah A, Chery C, Blanca-López N, et al. HLA-DRA variants predict penicillin allergy in genome-wide fine-mapping genotyping. J Allergy Clin Immunol. 2015;135(1):253-9.

6. Lee YH. Meta-analysis of genetic association studies. Ann Lab Med. 2015;35(3):283-7.

7. Wacholder S, Chanock S, Garcia-Closas M, El Ghormli L, Rothman N. Assessing the probability that a positive report is false: an approach for molecular epidemiology studies. J Natl Cancer Inst. 2004;96(6):434-42.

8. Ioannidis JP, Boffetta P, Little J, O'Brien TR, Uitterlinden AG, Vineis P, et al. Assessment of cumulative evidence on genetic associations: interim guidelines. Int J Epidemiol. 2008;37(1):120-32.

9. Romano A, Atanaskovic-Markovic M, Barbaud A, Bircher AJ, Brockow K, Caubet JC, et al. Towards a more precise diagnosis of hypersensitivity to beta-lactams - an EAACI position paper. Allergy. 2020;75(6):1300-15.

**Table S1** PRISMA 2020 checklist

| **Section and Topic** | **Item #** | **Checklist item** | **Location where item is reported** |
| --- | --- | --- | --- |
| **TITLE** | | |  |
| Title | 1 | Identify the report as a systematic review. | Page 1 |
| **ABSTRACT** | | |  |
| Abstract | 2 | See the PRISMA 2020 for Abstracts checklist. | Page 1-2 |
| **INTRODUCTION** | | |  |
| Rationale | 3 | Describe the rationale for the review in the context of existing knowledge. | Page 3 |
| Objectives | 4 | Provide an explicit statement of the objective(s) or question(s) the review addresses. | Page 4 |
| **METHODS** | | |  |
| Eligibility criteria | 5 | Specify the inclusion and exclusion criteria for the review and how studies were grouped for the syntheses. | Page 5 |
| Information sources | 6 | Specify all databases, registers, websites, organisations, reference lists and other sources searched or consulted to identify studies. Specify the date when each source was last searched or consulted. | Page 5 |
| Search strategy | 7 | Present the full search strategies for all databases, registers and websites, including any filters and limits used. | Supplement |
| Selection process | 8 | Specify the methods used to decide whether a study met the inclusion criteria of the review, including how many reviewers screened each record and each report retrieved, whether they worked independently, and if applicable, details of automation tools used in the process. | Page 5;  Supplement |
| Data collection process | 9 | Specify the methods used to collect data from reports, including how many reviewers collected data from each report, whether they worked independently, any processes for obtaining or confirming data from study investigators, and if applicable, details of automation tools used in the process. | Page 5-6 |
| Data items | 10a | List and define all outcomes for which data were sought. Specify whether all results that were compatible with each outcome domain in each study were sought (e.g. for all measures, time points, analyses), and if not, the methods used to decide which results to collect. | Page 5-6 |
|  | 10b | List and define all other variables for which data were sought (e.g. participant and intervention characteristics, funding sources). Describe any assumptions made about any missing or unclear information. | Page 5-6; supplement |
| Study risk of bias assessment | 11 | Specify the methods used to assess risk of bias in the included studies, including details of the tool(s) used, how many reviewers assessed each study and whether they worked independently, and if applicable, details of automation tools used in the process. | Page 6 |
| Effect measures | 12 | Specify for each outcome the effect measure(s) (e.g. risk ratio, mean difference) used in the synthesis or presentation of results. | Page 6 |
| Synthesis methods | 13a | Describe the processes used to decide which studies were eligible for each synthesis (e.g. tabulating the study intervention characteristics and comparing against the planned groups for each synthesis (item #5)). | Page 5-6; supplement |
|  | 13b | Describe any methods required to prepare the data for presentation or synthesis, such as handling of missing summary statistics, or data conversions. | Page 5-6; Supplement |
|  | 13c | Describe any methods used to tabulate or visually display results of individual studies and syntheses. | Supplement |
|  | 13d | Describe any methods used to synthesize results and provide a rationale for the choice(s). If meta-analysis was performed, describe the model(s), method(s) to identify the presence and extent of statistical heterogeneity, and software package(s) used. | Page 6, Supplement |
|  | 13e | Describe any methods used to explore possible causes of heterogeneity among study results (e.g. subgroup analysis, meta-regression). | Page 6; supplement |
|  | 13f | Describe any sensitivity analyses conducted to assess robustness of the synthesized results. |  |
| Reporting bias assessment | 14 | Describe any methods used to assess risk of bias due to missing results in a synthesis (arising from reporting biases). | supplement |
| Certainty assessment | 15 | Describe any methods used to assess certainty (or confidence) in the body of evidence for an outcome. | Page 6; supplement |
| **RESULTS** | | |  |
| Study selection | 16a | Describe the results of the search and selection process, from the number of records identified in the search to the number of studies included in the review, ideally using a flow diagram. | Page 8;  Figure 1 |
|  | 16b | Cite studies that might appear to meet the inclusion criteria, but which were excluded, and explain why they were excluded. | Page 8 |
| Study characteristics | 17 | Cite each included study and present its characteristics. | Page 8-9; Table S3,5 |
| Risk of bias in studies | 18 | Present assessments of risk of bias for each included study. | Supplement table S4 |
| Results of individual studies | 19 | For all outcomes, present, for each study: (a) summary statistics for each group (where appropriate) and (b) an effect estimate and its precision (e.g. confidence/credible interval), ideally using structured tables or plots. | Supplement table S6 |
| Results of syntheses | 20a | For each synthesis, briefly summarise the characteristics and risk of bias among contributing studies. | Page 9 |
|  | 20b | Present results of all statistical syntheses conducted. If meta-analysis was done, present for each the summary estimate and its precision (e.g. confidence/credible interval) and measures of statistical heterogeneity. If comparing groups, describe the direction of the effect. | Page 10; Table 3; S6 |
|  | 20c | Present results of all investigations of possible causes of heterogeneity among study results. | Page 10;  Table S7-S10 |
|  | 20d | Present results of all sensitivity analyses conducted to assess the robustness of the synthesized results. |  |
| Reporting biases | 21 | Present assessments of risk of bias due to missing results (arising from reporting biases) for each synthesis assessed. | NA |
| Certainty of evidence | 22 | Present assessments of certainty (or confidence) in the body of evidence for each outcome assessed. | Page 10;  Table S7-S10 |
| **DISCUSSION** | | |  |
| Discussion | 23a | Provide a general interpretation of the results in the context of other evidence. | Page 11-12 |
|  | 23b | Discuss any limitations of the evidence included in the review. | Page 13-14 |
|  | 23c | Discuss any limitations of the review processes used. | Page 13-14 |
|  | 23d | Discuss implications of the results for practice, policy, and future research. | Page 14-15 |
| **OTHER INFORMATION** | | |  |
| Registration and protocol | 24a | Provide registration information for the review, including register name and registration number, or state that the review was not registered. | Page 5 |
|  | 24b | Indicate where the review protocol can be accessed, or state that a protocol was not prepared. | Page 5 |
|  | 24c | Describe and explain any amendments to information provided at registration or in the protocol. | NA |
| Support | 25 | Describe sources of financial or non-financial support for the review, and the role of the funders or sponsors in the review. | Title page |
| Competing interests | 26 | Declare any competing interests of review authors. | Title page |
| Availability of data, code and other materials | 27 | Report which of the following are publicly available and where they can be found: template data collection forms; data extracted from included studies; data used for all analyses; analytic code; any other materials used in the review. | NA |

*From:*  Page MJ, McKenzie JE, Bossuyt PM, Boutron I, Hoffmann TC, Mulrow CD, et al. The PRISMA 2020 statement: an updated guideline for reporting systematic reviews. BMJ 2021;372:n71.

**Table S2.1** Search strategy in Ovid MEDLINE(R) ALL 1946 to September 15, 2022

| **Search** | **Query** | **Items Found** |
| --- | --- | --- |
| #1 | exp penicillin/ | 83729 |
| #2 | exp beta-lactam/ | 136324 |
| #3 | exp cephalosporin/ | 45479 |
| #4 | (penicillin* or beta-lactam* or cephalosporin*).mp. | 154353 |
| #5 | (penicillin* or aminopenicillin* or thiazolidine* or beta-lactam* or betalactam* or lactam* or cephalosporin* or aminocephalosporin* or carbapenem* or aztreonam* or monobactam*).ti,ab,kw,rn. | 188911 |
| #6 | or/1-5 | 231657 |
| #7 | exp gene/ | 819323 |
| #8 | exp genotype/ | 451324 |
| #9 | exp phenotype/ | 335928 |
| #10 | exp allele/ | 118087 |
| #11 | exp genome/ | 1065569 |
| #12 | exp genome-wide association study/ | 39111 |
| #13 | exp single nucleotide polymorphism/ | 134303 |
| #14 | exp human leukocyte antigen/ | 77368 |
| #15 | exp pharmacogenetics/ | 13203 |
| #16 | (genome-wide association study or GWAS or single nucleotide polymorphism* or SNP or human leukocyte antigen or HLA or haplotype or pharmacogenom* or pharmacogenet* or high-throughput nucleotide sequencing or immunochip*).ti,ab,kw. | 285150 |
| #17 | (gene* or genotyp* or phenotyp* or allele* or genom* or nucleotide) adj3 (polymorph* or variant* or mutat* or sequenc*).ti,ab,kw. | 589990 |
| #18 | or/7-17 | 2001316 |
| #19 | 6 and 18 | 19045 |
| #20 | exp allergy/ | 370182 |
| #21 | exp anaphylaxis/ | 22486 |
| #22 | exp hypersensitivity/ | 370182 |
| #23 | exp skin rash/ | 8921 |
| #24 | exp adverse drug reaction/ | 128556 |
| #25 | exp angio$edema/ | 6623 |
| #26 | exp Stevens-Johnson syndrome/ | 5894 |
| #27 | exp toxic epidermal necrolysis/ | 5894 |
| #28 | exp drug allergy/ | 49032 |
| #29 | (immediat* or delay* immunoglobulin or immun* or IgE or Gell-Coombs or skin or cutaneous) adj4 (hypersensitivity or reaction* or rash).ti,ab,kw. | 81816 |
| #30 | (allergy or anaphyla* angio$edema or bronchospasm or cardiovascular collapse or urticaria or adverse drug reaction* or ADR* or severe cutaneous adverse reaction or SCAR or acute generalized exanthematous pustulosis or AGEP or drug reaction with eosinophilia and systemic symptoms or DRESS or Stevens-Johnson syndrome or SJS or toxic epidermal necrolysis or TEN or SJS-TEN or lichenoid drug eruption or maculopapular exanthema or benign exanthema or h$emolytic a$nemia or thrombocytopenia or petechia or serum sickness or vessel vasculitis or arthus reaction*).ti,ab,kw. | 426316 |
| #31 | or/20-30 | 926964 |
| #32 | 19 and 31 | 619 |
| #33 | (news or newspaper article or comment or editorial or interview or letter or review or systematic review or case report or case series).pt. | 5439957 |
| #34 | 32 not 33 | 541 |
| #35 | limit 34 to human | 377 |

**Table S2.2 Search strategy in Embase via Elsevier 1966 to September 15, 2022**

| **Search** | **Query** | **Items Found** |
| --- | --- | --- |
| #1 | penicillin*/exp AND [embase]/lim | 144532 |
| #2 | beta-lactam*/exp AND [embase]/lim | 70815 |
| #3 | cephalosporin*/exp AND [embase]/lim | 65753 |
| #4 | (penicillin*:ti,ab,kw,rn OR aminopenicillin*:ti,ab,kw,rn OR thiazolidine*:ti,ab,kw,rn OR ‘beta-lactam*’:ti,ab,kw,rn OR ‘beta lactam*’:ti,ab,kw,rn OR betalactam*:ti,ab,kw,rn OR lactam*:ti,ab,kw,rn OR cephalosporin*:ti,ab,kw,rn OR aminocephalosporin*:ti,ab,kw,rn OR carbapenem*:ti,ab,kw,rn OR aztreonam*:ti,ab,kw,rn OR monobactam*:ti,ab,kw,rn) AND [embase]/lim | 146260 |
| #5 | #1 OR #2 OR #3 OR #4 | 259774 |
| #6 | gene*/exp AND [embase]/lim | 8610035 |
| #7 | genotype*/exp AND [embase]/lim | 488704 |
| #8 | phenotyp*/exp AND [embase]/lim | 905603 |
| #9 | allele*/exp AND [embase]/lim | 318017 |
| #10 | genom*/exp AND [embase]/lim | 1075565 |
| #11 | ‘genome-wide association stud*’/exp AND [embase]/lim | 57059 |
| #12 | ‘single nucleotide polymorphism*’/exp AND [embase]/lim | 206167 |
| #13 | ‘human leukocyte antigen’/exp AND [embase]/lim | 10 |
| #14 | Pharmacogenetic*/exp AND [embase]/lim | 34832 |
| #15 | (‘genome-wide association stud*’:ti,ab,kw OR GWAS:ti,ab,kw OR ‘single nucleotide polymorphism*’:ti,ab,kw OR SNP:ti,ab,kw OR ‘human leukocyte antigen’:ti,ab,kw OR HLA:ti,ab,kw OR haplotype*:ti,ab,kw OR pharmacogenom*:ti,ab,kw OR pharmacogenet*:ti,ab,kw OR ‘high-throughput nucleotide sequenc*’:ti,ab,kw OR immunochip*:ti,ab,kw) AND [embase]/lim | 377346 |
| #16 | ((gene*:ti,ab,kw OR genotyp*:ti,ab,kw OR phenotyp*:ti,ab,kw OR allele*:ti,ab,kw OR genom*:ti,ab,kw OR nucleotide:ti,ab,kw) AND (polymorph*:ti,ab,kw OR variant*:ti,ab,kw OR mutat*:ti,ab,kw OR sequenc*:ti,ab,kw)) AND [embase]/lim | 1744751 |
| #17 | #6 OR #7 OR #8 OR #9 OR #10 OR #11 OR #12 OR #13 OR #14 OR #15 OR #16 | 9161295 |
| #18 | #5 AND #16 | 21969 |
| #19 | allergy/exp AND [embase]/lim | 258708 |
| #20 | anaphyla*/exp AND [embase]/lim | 66508 |
| #21 | hypersensitivity/exp AND [embase]/lim | 659190 |
| #22 | ‘skin rash’/exp AND [embase]/lim | 131797 |
| #23 | ‘adverse drug reaction*’/exp AND [embase]/lim | 1243957 |
| #24 | angio?edema/exp AND [embase]/lim | 675 |
| #25 | ‘Stevens-Johnson syndrome’/exp AND [embase]/lim | 11063 |
| #26 | ‘toxic epidermal necrolysis’/exp AND [embase]/lim | 8813 |
| #27 | ‘drug allergy’/exp AND [embase]/lim | 70232 |
| #28 | ((immediat*:ti,ab,kw OR delay*:ti,ab,kw OR immunoglobulin:ti,ab,kw OR immun*:ti,ab,kw OR IgE:ti,ab,kw OR ‘Gell-Coombs’:ti,ab,kw OR skin:ti,ab,kw OR cutaneous) AND (hypersensitivity:ti,ab,kw OR reaction*:ti,ab,kw OR rash)) AND [embase]/lim | 405890 |
| #29 | (allergy:ti,ab,kw OR anaphyla*:ti,ab,kw OR angio?edema:ti,ab,kw OR bronchospasm:ti,ab,kw OR ‘cardiovascular collapse’:ti,ab,kw OR urticaria:ti,ab,kw OR ‘adverse drug reaction*’:ti,ab,kw OR ADR*:ti,ab,kw OR ‘severe cutaneous adverse reaction*’:ti,ab,kw OR SCAR:ti,ab,kw OR ‘acute generalized exanthematous pustulosis’:ti,ab,kw OR AGEP:ti,ab,kw OR ‘drug reaction with eosinophilia and systemic symptom*’:ti,ab,kw OR DRESS:ti,ab,kw OR ‘Stevens-Johnson syndrome’:ti,ab,kw OR SJS:ti,ab,kw OR ‘toxic epidermal necrolysis’:ti,ab,kw OR TEN:ti,ab,kw OR ‘SJS-TEN’:ti,ab,kw OR ‘lichenoid drug eruption’:ti,ab,kw OR ‘maculopapular exanthema’:ti,ab,kw OR ‘benign exanthema’:ti,ab,kw OR ‘h?emolytic a?nemia’:ti,ab,kw OR thrombocytopenia:ti,ab,kw OR petechia:ti,ab,kw OR ‘serum sickness’:ti,ab,kw OR ‘vessel vasculitis’:ti,ab,kw OR ‘arthus reaction*’:ti,ab,kw) AND [embase]/lim | 1058144 |
| #30 | #19 OR #20 OR #21 OR #22 OR #23 OR #24 OR #25 OR #26 OR #27 OR #28 OR #29 | 2856752 |
| #31 | #18 AND #30 | 1246 |
| #32 | (news:it OR ‘newspaper article’:it OR comment:it OR editorial:it OR interview:it OR  letter:it OR review:it OR ‘systematic review’:it OR ‘case report’:it OR ‘case series’:it) AND [embase]/lim | 3744912 |
| #33 | #31 NOT #32 | 1048 |

**Table S2.3 Search strategy in PubMed, From Inception to September 15, 2022**

| **Search** | **Query** | **Items Found** |
| --- | --- | --- |
| #1 | beta-lactam*[Pharmacological Action] | 11180 |
| #2 | ((penicillin[MeSH Terms]) OR (beta-lactam[MeSH Terms])) OR (beta lactamase, cephalosporin[MeSH Terms]) | 136405 |
| #3 | penicillin*[Title/Abstract] OR aminopenicillin*[Title/Abstract] OR thiazolidine*[Title/Abstract] OR "beta-lactam*"[Title/Abstract] OR betalactam*[Title/Abstract] OR lactam*[Title/Abstract] OR cephalosporin*[Title/Abstract] OR aminocephalosporin*[Title/Abstract] OR carbapenem*[Title/Abstract] OR aztreonam*[Title/Abstract] OR monobactam*[Title/Abstract] | 143122 |
| #4 | #1 OR #2 OR #3 | 211957 |
| #5 | (((((((associations, genotype phenotype[MeSH Terms]) OR (allele[MeSH Terms])) OR (association study, genome wide[MeSH Terms])) OR (single nucleotide polymorphism[MeSH Terms])) OR (antigens, human leukocyte[MeSH Terms])) OR (analyses, genetic linkage[MeSH Terms])) OR (pharmacogenetics[MeSH Terms])) OR (pharmacogenomics[MeSH Terms]) | 375521 |
| #6 | “genome-wide association stud*”[Title/Abstract] OR GWAS[Title/Abstract] OR “single nucleotide polymorphism*”[Title/Abstract] OR SNP[Title/Abstract] OR “human leukocyte antigen”[Title/Abstract] OR HLA[Title/Abstract] OR haplotype*[Title/Abstract] OR pharmacogenom*[Title/Abstract] OR pharmacogenet*[Title/Abstract] OR “high-throughput nucleotide sequenc*”[Title/Abstract] OR immunochip*[Title/Abstract] | 306311 |
| #7 | (gene*[Title/Abstract] OR genotyp*[Title/Abstract] OR phenotyp*[Title/Abstract] OR allele*[Title/Abstract] OR genom*[Title/Abstract] OR nucleotide[Title/Abstract]) AND (polymorph*[Title/Abstract] OR variant*[Title/Abstract] OR mutat*[Title/Abstract] OR sequenc*[Title/Abstract]) | 1656650 |
| #8 | #5 OR #6 OR #7 | 1874908 |
| #9 | #4 AND #8 | 17411 |
| #10 | ((((((((((allergy and immunology[MeSH Terms]) OR (allergy, drug[MeSH Terms])) OR (anaphylactic reaction[MeSH Terms])) OR (anaphylactic shock[MeSH Terms])) OR (anaphylaxis[MeSH Terms])) OR (skin rash[MeSH Terms])) OR (adverse drug reaction[MeSH Terms])) OR (angioedema[MeSH Terms])) OR (stevens johnson syndrome[MeSH Terms])) OR (toxic epidermal necrolysis[MeSH Terms])) OR (drug allergy[MeSH Terms]) | 177628 |
| #11 | (immediat*[Title/Abstract] OR delay*[Title/Abstract] OR immunoglobulin[Title/Abstract] OR immun*[Title/Abstract] OR IgE[Title/Abstract] OR “Gell-Coombs”[Title/Abstract] OR skin[Title/Abstract] OR cutaneous[Title/Abstract]) AND (hypersensitivity[Title/Abstract] OR reaction*[Title/Abstract] OR rash[Title/Abstract]) | 330424 |
| #12 | allergy[Title/Abstract] OR anaphyla*[Title/Abstract] OR angio?edema[Title/Abstract] OR bronchospasm[Title/Abstract] OR “cardiovascular collapse”[Title/Abstract] OR urticaria[Title/Abstract] OR “adverse drug reaction*”[Title/Abstract] OR ADR*[Title/Abstract] OR “severe cutaneous adverse reaction*”[Title/Abstract] OR SCAR[Title/Abstract] OR “acute generalized exanthematous pustulosis”[Title/Abstract] OR AGEP[Title/Abstract] OR “drug reaction with eosinophilia*”[Title/Abstract] OR DRESS[Title/Abstract] OR “Stevens-Johnson syndrome”[Title/Abstract] OR SJS[Title/Abstract] OR “toxic epidermal necrolysis”[Title/Abstract] OR TEN[Title/Abstract] OR “SJS-TEN”[Title/Abstract] OR “lichenoid drug eruption”[Title/Abstract] OR “maculopapular exanthema”[Title/Abstract] OR “hemolytic anemia”[Title/Abstract] OR thrombocytopenia[Title/Abstract] OR petechia[Title/Abstract] OR “serum sickness”[Title/Abstract] OR “vessel vasculitis”[Title/Abstract] OR “arthus reaction*”[Title/Abstract] | 620193 |
| #13 | #10 OR #11 OR #12 | 1038220 |
| #14 | #9 AND #13 | 678 |
| #15 | Filters applied: Humans | 462 |

**Table S2.4 Search strategy in Cochrane Library (CENTRAL), From Inception to September 15, 2022**

| **Search** | **Query** | **Items Found** |
| --- | --- | --- |
| #1 | MeSH descriptor: [Penicillins] explode all trees | 5895 |
| #2 | MeSH descriptor: [beta-Lactams] explode all trees | 9880 |
| #3 | MeSH descriptor: [Cephalosporins] explode all trees | 4498 |
| #4 | (penicillin* OR aminopenicillin* OR thiazolidine* OR "beta-lactam*" OR betalactam* OR lactam* OR cephalosporin* OR aminocephalosporin* OR carbapenem* OR aztreonam* OR monobactam*):ti,ab,kw | 9951 |
| #5 | #1 OR #2 OR #3 OR #4 | 15547 |
| #6 | MeSH descriptor: [Genes] explode all trees | 1726 |
| #7 | MeSH descriptor: [Genotype] explode all trees | 4810 |
| #8 | MeSH descriptor: [Phenotype] explode all trees | 1561 |
| #9 | MeSH descriptor: [Alleles] explode all trees | 753 |
| #10 | MeSH descriptor: [Genome] in all MeSH products | 2150 |
| #11 | MeSH descriptor: [Genome-Wide Association Study] explode all trees | 192 |
| #12 | MeSH descriptor: [Polymorphism, Genetic] explode all trees | 3197 |
| #13 | MeSH descriptor: [Polymorphism, Single Nucleotide] explode all trees | 1437 |
| #14 | MeSH descriptor: [HLA Antigens] explode all trees | 697 |
| #15 | MeSH descriptor: [Pharmacogenomic Testing] explode all trees | 72 |
| #16 | (“genome-wide association stud*” OR GWAS OR “single nucleotide polymorphism*” OR SNP OR “human leukocyte antigen” OR HLA OR haplotype* OR pharmacogenom* OR pharmacogenet* OR “high-throughput nucleotide sequenc*” OR immunochip*):ti,ab,kw | 11069 |
| #17 | (gene* OR genotyp* OR phenotyp* OR allele* OR genom* OR nucleotide):ti,ab,kw AND (polymorph* OR variant* OR mutat* OR sequenc*):ti,ab,kw | 33395 |
| #18 | #6 OR #7 OR #8 OR #9 OR #10 OR #11 OR #12 OR #13 OR #14 OR #15 OR #16 OR #17 | 42700 |
| #19 | #5 AND #18 | 407 |
| #20 | MeSH descriptor: [Hypersensitivity] explode all trees | 22069 |
| #21 | MeSH descriptor: [Drug Hypersensitivity] explode all trees | 1020 |
| #22 | MeSH descriptor: [Anaphylaxis] explode all trees | 198 |
| #23 | MeSH descriptor: [Exanthema] explode all trees | 226 |
| #24 | MeSH descriptor: [Drug-Related Side Effects and Adverse Reactions] explode all trees | 3885 |
| #25 | MeSH descriptor: [Angioedema] explode all trees | 241 |
| #26 | MeSH descriptor: [Stevens-Johnson Syndrome] explode all trees | 29 |
| #27 | (immediat* OR delay* OR immunoglobulin OR immun* OR IgE OR “Gell-Coombs” OR skin OR cutaneous):ti,ab,kw AND (hypersensitivity OR reaction* OR rash):ti,ab,kw | 43587 |
| #28 | (allergy OR anaphyla* OR angio?edema OR bronchospasm OR “cardiovascular collapse” OR urticaria OR “adverse drug reaction*” OR ADR* OR “severe cutaneous adverse reaction*” OR SCAR OR “acute generalized exanthematous pustulosis” OR AGEP OR “drug reaction with eosinophilia and systemic symptom*” OR DRESS OR “Stevens-Johnson syndrome” OR SJS OR “toxic epidermal necrolysis” OR TEN OR “SJS-TEN” OR “lichenoid drug eruption” OR “maculopapular exanthema” OR “benign exanthema” OR “h?emolytic a?nemia” OR thrombocytopenia OR petechia OR “serum sickness” OR “vessel vasculitis” OR “arthus reaction*”):ti,ab,kw | 151148 |
| #29 | #20 OR #21 OR #22 OR #23 OR #24 OR #25 OR #26 OR #27 OR #28 | 188912 |
| #30 | #19 AND #29 | 74 |
| #31 | #19 AND #29 in Trials | 70 |

**Table S2.5** Search strategy in Scopus, From Inception to September 15, 2022

| **Search** | **Query** | **Items Found** |
| --- | --- | --- |
| #1 | TITLE-ABS-KEY (penicillin* OR aminopenicillin* OR thiazolidine* OR "beta-lactam*" OR betalactam* OR lactam* OR cephalosporin* OR aminocephalosporin* OR carbapenem* OR aztreonam* OR monobactam*) | 356987 |
| #2 | TITLE-ABS-KEY (gene* OR genotyp* OR phenotyp* OR allele* OR “genome-wide association stud*” OR GWAS OR “single nucleotide polymorphism*” OR SNP OR “human leukocyte antigen” OR HLA OR haplotype* OR pharmacogenom* OR pharmacogenet* OR “high-throughput nucleotide sequenc*” OR immunochip*) | 16812024 |
| #3 | TITLE-ABS-KEY ((gene* OR genotyp* OR phenotyp* OR allele* OR genom* OR nucleotide) AND (polymorph* OR variant* OR mutat* OR sequenc*)) | 3362469 |
| #4 | #2 OR #3 | 16927730 |
| #5 | #1 AND #4 | 104047 |
| #6 | TITLE-ABS-KEY ((immediat* OR delay* OR immunoglobulin OR immun* OR IgE OR “Gell-Coombs” OR skin OR cutaneous) AND (hypersensitivity OR reaction* OR rash)) | 1158149 |
| #7 | TITLE-ABS-KEY (hypersensitivity OR allergy OR anaphyla* OR angio?edema OR bronchospasm OR “cardiovascular collapse” OR urticaria OR “adverse drug reaction*” OR ADR* OR “severe cutaneous adverse reaction*” OR SCAR OR “acute generalized exanthematous pustulosis” OR AGEP OR “drug reaction with eosinophilia and systemic symptom*” OR DRESS OR “Stevens-Johnson syndrome” OR SJS OR “toxic epidermal necrolysis” OR TEN OR “SJS-TEN” OR “lichenoid drug eruption” OR exanthema OR “maculopapular exanthema” OR “benign exanthema” OR “h?emolytic a?nemia” OR thrombocytopenia OR petechia OR “serum sickness” OR “vessel vasculitis” OR “arthus reaction*”) | 2356931 |
| #8 | #6 OR #7 | 3248056 |
| #9 | #5 AND #8 | 12465 |
| #10 | TITLE-ABS-KEY (animal OR in vivo OR in vitro) | 2459334 |
| #11 | #9 AND NOT #10 | 10850 |
| #12 | #11 AND NOT ( TITLE-ABS-KEY ( animal OR in AND vivo OR in AND vitro ) ) AND ( EXCLUDE ( DOCTYPE , "le" ) OR EXCLUDE ( DOCTYPE , "sh" ) OR EXCLUDE ( DOCTYPE , "no" ) OR EXCLUDE ( DOCTYPE , "ed" ) OR EXCLUDE ( DOCTYPE , "re" ) OR EXCLUDE ( DOCTYPE , "cp" ) OR EXCLUDE ( DOCTYPE , "ch" ) OR EXCLUDE ( DOCTYPE , "tb" ) OR EXCLUDE ( DOCTYPE , "er" ) ) | 8285 |

**Table S2.6** Search strategy in CINAHL, From Inception to September 15, 2022

| **Search** | **Query** | **Items Found** |
| --- | --- | --- |
| #1 | MJ (penicillin OR beta lactam antibiotics OR cephalosporins) | 1167 |
| #2 | AB (penicillin* OR aminopenicillin* OR thiazolidine* OR "beta-lactam*" OR betalactam* OR lactam* OR cephalosporin* OR aminocephalosporin* OR carbapenem* OR aztreonam* OR monobactam*) | 11180 |
| #3 | S1 OR S2 | 11876 |
| #4 | MJ (genetic OR genes OR genotype OR phenotype OR allele OR genome OR genome-wide association studies OR GWAS OR polymorphism, genetic OR single nucleotide polymorphism OR SNP OR human leucocyte antigen OR pharmacogenomics OR pharmacogenetics) | 82317 |
| #5 | AB (“genome-wide association stud*” OR GWAS OR “single nucleotide polymorphism*” OR SNP OR “human leukocyte antigen” OR HLA OR haplotype* OR pharmacogenom* OR pharmacogenet* OR “high-throughput nucleotide sequenc*” OR immunochip*) | 24812 |
| #6 | AB ((gene* OR genotyp* OR phenotyp* OR allele* OR genom* OR nucleotide) AND (polymorph* OR variant* OR mutat* OR sequenc*)) | 98374 |
| #7 | S4 OR S5 OR S6 | 159563 |
| #8 | S3 AND S7 | 1167 |
| #9 | MJ (allergy or allergies OR hypersensitivity reactions OR anaphylaxis OR drug allergy OR skin rash OR adverse drug reactions OR angioedema OR stevens-johnson syndrome OR toxic epidermal necrolysis) | 6079 |
| #10 | AB ((immediat* OR delay* OR immunoglobulin OR immun* OR IgE OR “Gell-Coombs” OR skin OR cutaneous) AND (hypersensitivity OR reaction* OR rash)) | 30233 |
| #11 | AB (allergy OR anaphyla* OR angio?edema OR bronchospasm OR “cardiovascular collapse” OR urticaria OR “adverse drug reaction*” OR ADR* OR “severe cutaneous adverse reaction*” OR SCAR OR “acute generalized exanthematous pustulosis” OR AGEP OR “drug reaction with eosinophilia and systemic symptom*” OR DRESS OR “Stevens-Johnson syndrome” OR SJS OR “toxic epidermal necrolysis” OR TEN OR “SJS-TEN” OR “lichenoid drug eruption” OR “maculopapular exanthema” OR “benign exanthema” OR “h?emolytic a?nemia” OR thrombocytopenia OR petechia OR “serum sickness” OR “vessel vasculitis” OR “arthus reaction*”) | 390250 |
| #12 | S9 OR S10 OR S11 | 416722 |
| #13 | S8 AND S12 | 156 |
| #14 | MW (animal OR in vivo OR in vitro) | 185382 |
| #15 | S13 NOT S14 | 138 |
| #16 | Expanders: Apply equivalent subjects  Source Types: Academic Journals | 137 |

**Table S3** Characteristics of included candidate gene studies of genetic association of Beta-lactams (BL) allergy at single variant association

| **Author, year (Study site)** | **Causative drugs (%)** | **Immediate/non-immediate (%/%)** | **Clinical symptoms (%)** | **Gene (variant)**  *=significant | **Case phenotype^a^** | **Control phenotype^b^** | **Sample size, case/ control** | **Significant effect estimate, (95%CI)^c^** | **Functional validation** | **STREGA score** |
| --- | --- | --- | --- | --- | --- | --- | --- | --- | --- | --- |
| Qiao et al., 2004  (China, Zhengzhou University) | penicillin G (80.4);  ampicillin (8.9);  amoxicillin (5.1) | 65.2/ 34.8 | Shock (22.2),  Urticaria (43.0),  Others (13.3) | FceRIb (E237G) | Positive skin test or show clinical symptoms | No history of allergic diseases | 158/87 | NA | - Skin test  - RAST | 10 |
| Qiao et al., 2005  (China, Zhengzhou University) | penicillin G (80.12),  ampicillin (8.3),  amoxicillin (5.13) | 64.7/ 35.3 | NA | IL-4Ra (Q576R)* | Positive skin test or show clinical symptoms | No history of allergic diseases and negative functional test | 156/87 | NA | - Skin test  - RAST  - Serum level IL-4, IL-13, IFN-g by ELISA | 9 |
| Yang et al., 2005  (China, Zhengzhou University) | penicillin G (82.3),  ampicillin (5.7),  amoxicillin (12.0) | 44.3/ 55.7 | Shock (22.2),  Urticaria (43.0),  Others (13.3) | IL-13 (R130Q);  IL-4-IL13 (SNP-3);  IL-4-IL-13 (SNP4); | Positive skin test or show clinical symptoms | No history of allergic diseases and negative functional test | 158/89 | NA | - Skin test  - RAST  - Serum level IL-4, IL-13, IFN-g by ELISA | 10 |
| Guglielmi et al., 2006  (France, University Hospital of Montpellier) | Penicillin G,  Amoxicillin,  Ampicillin,  Other BL | NA | NA | IL-4 (-589)  IL-4 (-34)  IL-13 (R130Q)  IL-13 (-1512)  IL-13 (-1112)  IL-4RA (I75V)*  IL-4RA (Q576R)  IL-10 (-1082)*  IL-10 (-819)*  IL-10 (-592)  IL-21R (-83)  STAT6 (in 18)  FceRIb (E237G)  IFNGR1 (V14M)  IFNGR2 (Q64R)  IL-10 (Haplotype) | Positive skin prick test or positive provocation test  (Immediate reaction) | Negative skin test or provocation test | 44/44 | - IL-4Ra (I50V), OR=5.4, (CI:  1.16–27.7) only in atopic female  - IL-10 (-1082), OR=17.5, (CI: 1.26-533.1)  - IL-10 (-819), OR=17.5, (CI: 1.26-533.1) | - Skin prick test to BL  - Drug provocation test | 11 |
| Gueant-Rodriguez et al., 2006  (Italy, C.I. Columbus and Oasi  Maria S.S. institute) | Penicillins (59), Cephalosporins (41) | Immediate only | NA | IL13 (R130Q)*  IL13 (–1055 C> T)*  IL4RA (I50V)*  IL4RA (S478P)  IL4RA (Q551R)* | Had allergic reaction to BL and positive functional validation | No history of allergic diseases | 210/265 | NS | - Skin test  - Serum specific IgE | 13 |
| Qiao et al., 2007  (China, Zhengzhou University) | Penicillins (90.20), Ampicillin (1.96), Amoxicillin (5.88),  Other BL (1.96) | NA | Shock (13.7),  Urticaria (24.5),  others(7.8) | IL10 (-1082 G/A)*  IL10 (–819 C/T) | Positive skin test or had allergic history | No history of allergic diseases and negative functional test | 102/86 | NA | - Skin test  - RAST  - Serum level IL-10 by ELISA | 10 |
| Apter et al., 2008  (USA, multicenter) | Penicillins and its derivatives  (Amoxicillin, Augmention, Ampicillin, Dicloxacin, Ticarcillin, Imipenem) | NA | NA | IL-4 (rs2070874)*  IL-4 (rs2243291)  IL-4 (rs12186803)  IL-4 (rs10062446)*  IL-4 (rs11740584)*  LACTB (rs1126309)  LACTB (rs1566660)  LACTB (rs2652820) LACTB (rs2729835) LACTB (rs34536322)  IL-10 (rs1800871)  IL-10 (rs1800872)  IL-4R (rs1805010) | Allergist-identified patients with history of penicillin allergy based on patient-assessed questionnaire | Tolerated to penicillins within 5 years | 23/39 | - IL-4 (rs2070874), OR= 3.33 (CI: 1.09, 10.21)  - IL-4 (rs10062446), OR= 3.61 (CI: 1.21, 10.71)  - IL-4 (rs11740584), OR= 4.08 (1.35, 12.30) | None | 16 |
| Gao et al., 2008  ( China, Zhengzhou University) | Penicillins | 70.8/29.2 | Shock (27.8),  Urticaria (32.6),  Others (15.3) | IFNR1 (Val14Met)* | Positive skin test or show clinical symptoms | No history of allergic diseases and negative functional test | 144/88 | NA | - Skin test  - RAST  - Serum level IL-14, IL-13 by ELISA  - Serum specific IgG | 8 |
| Gueant-Rodriguez et al., 2008  (Italy, (Italy, C.I. Columbus and Oasi  Maria S.S. institute) | Penicillins (56.2),  Cephalosporin (43.8 | Immediate only | Urticaria (16.0),  Angioedema or urticaria (13.0),  Anaphylactic shock (71.0) | TNFA (–308G > A)* | Had history of BL allergy and positive functional test | No history of drug allergy and immunological diseases | 169/260 | OR= 4.29 (CI: 1.11–16.5) | - Skin test  - Total serum IgE | 12 |
| Huang et al., 2009  (China, Zhengzhou University) | penicillin G; phenoxomethyl penicillin; ampicillin; amoxicillin | 89.3/10.7 | Shock (12.8),  Urticaria (18.2),  Others (6.6) | IL-4Ra (Q576R)*;  IL-4Ra (I75V) | Positive skin test or show clinical symptoms | No history of allergic diseases and negative functional test | 242/220 | NA | - Skin test  - RAST | 13 |
| Zhang et al., 2009  (China, Zhengzhou University) | Penicillins | NA | NA | IL-4 (C-589T) | Positive skin test or show clinical symptoms | No history of allergic diseases and negative functional test | 50/55 | NA | - Skin prick test  - RAST | 10 |
| Yue Wen Zhang et al., 2010  (China, Zhengzhou University) | Penicillins | NA | NA | IL-4 Rα (Q576R)  FcɛRIβ (E237G) | Positive skin test or show clinical symptoms | No history of allergic diseases and negative functional test | 51/53 | NA | - Skin prick test  - RAST | 10 |
| Zhang et al., 2010  (China, Zhengzhou University) | Penicillins | NA | NA | IL-13 (+2044G/A) | Positive skin test or show clinical symptoms | No history of allergic diseases and negative functional test | 51/20 | NA | - Skin prick test  - RAST | 10 |
| Ming et al., 2011  (China, Zhengzhou University) | Penicillins and its derivatives (penicillin G, phenoxymethylpenicillin, ampicillin, amoxicillin etc.) | 55.9/44.1 | Shock (13.4),  Dyspnea (16.2),  Urticaria (32.5),  Others (6.6) | IL-18 (-607 A/C)*  IL-18 (-137 G/C)* | Positive skin test or show clinical symptoms | No history of allergic diseases , negative functional test and no family history of allergy | 606/614 | - IL-18 (-607 A/C), OR=1.51 (CI: 1.28, 1.78)  - IL-18 (-137 G/C), OR=1.79 (CI: 1.40, 2.28) | - Skin test  - serum level IL-18 by ELISA | 12 |
| Chen et al., 2012  (China, Shanghai Institute for Biological  Sciences) | Penicillins | NA | NA | IFIH1 (rs1990760)  IFIH1 (rs3747517) | Positive skin test and history of penicillin allergy | Healthy controls without autoimmune disease or chronic disease | 175/1,101 | NA | - Skin test | 14 |
| Cornejo-García et al., 2012  (Spain,  Carlos Haya Hospital) | Culprit drug (54.7);  Penicilloyl polylysine (15.9);  Minor determinants (14.4);  Benzylpenicillin (5.9);  Amoxicillin (48.5) | Immediate only | Urticaria and/or angioedema (20.6),  Anaphylaxis (58.2),  Anaphylactic shock (21.2),  Allergic rhinitis (7.5),  Asthma (3.8),  Allergic rhinitis and asthma (4.7) | IL13 (R130Q)  TNFA (-308G>A)  IL4RA (I50V)  IL4RA (S478P)  IL4RA (Q551R);  IL4 (C-33T);  STAT6 (6613C>T);  LACTB (1523A>G) | Clinical symptoms or positive functional test | Volunteer from the same area | 340/340 | NA | - Skin test  - Drug provocation  - Total serum IgE  - RAST | 11 |
| Huang et al., 2012  (China, Zhengzhou University) | penicillin G; phenoxomethyl penicillin; ampicillin; amoxicillin; etc. | 19.0/10.7 | Shock (4.96),  Urticaria (18.18),  others (6.61) | STAT6 (2SNP3)* | Positive skin test or show clinical symptoms | No history of allergic diseases and negative functional test | 242/220 | NA | - Skin test  - RAST | 10 |
| Nam et al., 2012  (Korea, Ajou University Hospital) | Cephalosporin | NA | NA | FceR1b (-109T >  C)*,  FceR1b (-237A>G)*,  IL-4 (-33T>C)*; | Positive specific IgE after sensitization | Negative specific IgE after sensitization | 153/86 | - FceR1b (-109T>C, TT vs CT or CC),OR=3.55 (CI:1.32,9.53);  - FceR1b (-237A>G, AA vs AG or GG), OR=6.25 (CI:1.31,29.9)  - IL-4 (-33T>C, TT or CT vs CC), OR=5.78 (CI: 0.04,0.86) | - Skin-prick tests  - Measurement of total IgE  - Specific serum IgE and IgG | 13 |
| Bursztejn et al., 2013  (Developmental study)  (Italy) | Penicillins (75.0),  Cephalosporin (25.0) | 57.1/42.9 | NA | NOD2 (rs2066844),  NOD2 (rs2066845)*,  NOD2 (rs5743293),  NOD1 (rs2907749) | Positive functional test | Volunteers without allergic disease | 368/368 | NA | - Skin test  - Total serum IgE | 12 |
| Bursztejn et al., 2013 (Validation study; Spain) | Penicillins (93.9),  Cephalosporin, (6.1) | Immediate only | NA | NOD2 (rs2066844),  NOD2 (rs2066845),  NOD2 (rs5743293)* | Positive functional test | Volunteers without allergic disease | 387/326 | NA | - Skin test  - Total serum IgE | - |
| Li, 2015  (China, Lanzhou University Second Hospital) | Beta-lactams | Immediate only | NA | L-10 (rs1800871),  IL-10 (rs1800872),  IL-10 (rs1800896),  IL-13 (rs20541)*,  IL-13 (rs1881457,)  IL-13 (rs1800925),  IL-4Rα (rs1801275),  FcεRIβ (rs569108),  IFNGR2 (rs9808753),  CYP3A4 (rs2242480); | Had history of BL allergy and positive skin test | No history of allergic diseases and negative functional test | 64/30 | - CYP3A4 (rs2242480, CT vs CC), OR=0.167 (CI: 0.03, 0.87) | -Skin test | 13 |
| Cornejo-García et al., 2016  (Developmental study)  (Spain, Regional University Hospital of Malaga) | Amoxicillin (79.0),  Benzilpenicillin (12.7),  Amoxicillin-clavulanic acid (1.5),  Cephalosporins (4.8)  Other 0 (0) | Immediate only | Anaphylaxis (78.7),  Urticaria/angioedema,(21.3) | LGALS3 (rs4644),  LGALS3 (rs4652),  LGALS3 (rs11125)* | Diagnosis based on the standard protocol of the Europeans Network for drug allergy | No history of drug allergy and immunological diseases | 395/310 | - LGALS3 (rs11125), OR= 3.98 (CI: 2.70–6.00) | -Skin test  -RAST  - Total serum IgE | 14 |
| Cornejo-García et al., 2016  (Validation study; Italy, C.I. Columbus and Oasi  Maria S.S. institute) | Amoxicillin (42.0),  Benzilpenicillin (5.6),  Amoxicillin-clavulanic acid (10.2),  Cephalosporins (25.6),  Other (16.3) | Immediate only | Anaphylaxis (65.3),  Urticaria/ angioedema (34.7) | LGALS3 (rs11125)* | Diagnosis based on the standard protocol of the Europeans Network for drug allergy | No history of drug allergy and immunological diseases | 198/339 | - LGALS3 (rs11125), OR= 5.1 (CI: 3.58–7.31) | -Skin test  -RAST  - Total serum IgE | 14 |
| Khadhim et al., 2018  (Iraq, AL-Diwaniya Teaching  Hospital.) | Beta-lactams | NA | NA | IL-4Rα (Q576R)* | Had history of BL allergy and positive skin test | No history of BL allergy | 50/50 | OR=1.14 (CI: 0.17, 0.95), P=0.035 | -Skin test  -Serum IL-4 level by ELISA | 12 |
| Amo et al., 2019  (Spain) | Beta lactams | NA | NA | FCER1G (rs36233990),  FCER1G (rs2070901),  GC (rs3733359) | Diagnosed by allergists based on functional test | Healthy controls | 561/406 | FCER1G (rs36233990), OR=1.67 (CI:1.53-1.76)  FCER1G (rs2070901), OR=0.73 (CI:0.55-0.96) | - Skin prick test - Intradermal test - Provocation test - Specific IgE | 16 |
| Ksouri et al., 2019  (Algeria, Batna University) | Penicillins,  Cephalosporins | Immediate only | Urticaria (9.55),  Anaphylactic shock (7.54),  Asthma (9.55) | IL4RA (S478P),  IL-4Rα (I50V)*,  IL4RA (Q551R),  IL13 (R130Q)* | Positive functional test | No history of drug allergy and immunological diseases | 199/99 | IL13 (R130Q), OR= 3.56 (CI: 1.78–7.12) | - Skin test  - Total serum IgE | 12 |
| Rivera-Reigada et al., 2020  (Spain, University Hospital of Salamanca) | Bencylpenicillin (3.1),  Amoxicillin (79.5),  Cloxacillin, (2.1)  Cephalosporins (9.2),  Amoxicillin-clavulanate (3.1),  Unknown (3) | Immediate only | Urticaria/ angioedema (54.1),  Anaphylaxis (32.5),  Rash (9.4),  Nonspecific reactions (4) | IL1R (pst1+1970)*,  IFNG (+874),  IL4 (–33)* | Diagnosed with immediate BL allergy based on functional test | Negative functional test and tolerated full dose of a BL | 19/77 | NA | - Skin test  - Serum specific IgE  - Provocation test | 10 |
| Yang et al., 2006  (China, Zhengzhou University) | penicillin G (60.74),  ampicillin (4.01),  amoxicillin (2.58) | 79.8/20.2 | Shock (13.71),  Urticaria (27.82),  Others (12.90) | HLA-DRB1, HLA-DRB3, HLA-DRB4, HLA-DRB5 ( DR1.1, DR3, DR4, DR7, DR8, DR9*, DR10*, DR11, DR12, DR13.1, DR14.1, DR15, DR16,DR17, DR18, DR51, DR52 and DR53) | Positive skin test or show clinical symptoms | No history of allergic reaction and HLA related disease | 77/87 | NA | -Skin test  -RAST | 11 |
| Singvicharn et al., 2021  (Thailand, Ramathibodi Hospital) | Amoxicillin (58.3), Cloxacillin (8.3), ceftriaxone (8.3), Amoxicillin-clavulanate (16.7), meropenam (4.2), Ampi/sulbac (4.2) | 58.3/41.7 | SJS (4.2),  DRESS (4.2),  Urticaria/ angioedema (58.3),  Anaphylaxis (8.3),  Maculopapular eruption (20.8),  Fixed drug eruption (4.2) | HLA-A,  HLA-B,  HLA-C,  HLAA-DRB1 | Had history of BL allergy and confirmed diagnosis with functional test or report of SCAR | Healthy Thai children with no allergic reaction to BL provocation | 24/93 | - HLA-C (C*04:06), OR=13.4 (CI: 1.3, 132.71)  - HLA-C (C*08:01), OR=4.83 (CI: 1.93, 16.7) | - Skin prick test  - Intradermal skin test  - Drug provocation test | 15 |

a = maximum number of cases among all variants; b = maximum number of controls among all variants; c = considered only significant individual variant, not involve combination; * = significant due to chi-square or Fisher’s exact test for association; IR = Immediate reaction; NIR = non-immediate reaction; NA = data of effect size not available; NS = not significant; RAST = Radioallergosorbent test using antigenic determinant paper disc to test IgE

**Table S4** Level of allergic validation evidence according to allergic test procedure

| **Level of allergic assessment test** | **List of studies** |
| --- | --- |
| **Level 1**  Studies remarked No in vitro/ in vivo test, only history and questionnaires | Apter et al., 2008 |
| **Level 2**  Studies that Performing Skin test and/or serum IgE level and/or ELISA | Qiao et al., 2004; Qiao et al., 2005; Yang et al., 2005; Gueant et al., 2006; Qiao et al., 2007; Gao et al., 2008; Huang et al., 2009; Zhang et al., 2009; Zhang et al., 2010; Zhang et al., 2010; Ming et al., 2011; Chen et al., 2012; Huang et al., 2012; Nam, 2012; Bursztein et al., 2013; Li et al., 2015; Cornejo-Garcia et al., 2016; Khadhim et al., 2018; Ksouri et al., 2019; Gueant et al., 2008; Yang et al., 2006 |
| **Level 3**  Addressing drug provocation test | Gouglemi et al., 2006; Cornejo-Garcia et al., 2012; Amo et al., 2019; Riviera-Reigada et al., 2020; Singvicharn et al., 2021 |

**Table S5** Characteristics of included GWAS of Beta-lactams (BL) allergy

| **Author, year (Study site)** | **Causative drugs (%)** | **Immediate/ non-immediate (%/%)** | **Clinical symptoms (%)** | **Genotyped case (N) , phenotype** | **Genotyped control (N), phenotype** | **Sample size Case/ control** | **Discovered variant** | **Functional validation** | **STREGA score** |
| --- | --- | --- | --- | --- | --- | --- | --- | --- | --- |
| Gueant et al., 2015  (Discovery study; Spain) | Amoxicillin (85.4),  Cephalosporin (5.8),  Other BL (8.8) | Immediate only | Shock (50.7) | Age 16-60 and had history of immediate reaction to BL | No allergic diseases | 387/1,124 | - ZNF300 (rs4958427), OR=1.85 (P=1.8E-8);  - C5 (rs17612), OR=0.27 (P=4.4E-7);  - HLA-DRA|HLA-DRB5 (rs7754768), OR=0.63 (P=1.4E-6);  - HLA-DRA|HLA-DRB5 (rs9268832), OR=0.64 (P=4.1E-6);  - HLA-DRA (rs7192), OR=0.65 (P=8.1E-6);  - HLA-DRA|HLA-DRB5 (rs2227139), OR=0.65 (P=8.2E-6);  - HLA-DRA|HLA-DRB5 (rs2213586), OR=0.66 (P=1.4E-5);  - HLA-DRA|HLA-DRB5 (rs2213585), OR=0.66 (P=1.5E-5);  - HLA-DRA (rs7195), OR=0.66 (P=2.0E-5);  - HLA-DRA (rs8084), OR=0.68 (P=4.7E-5) | - Skin test  - Total serum IgE level  - Drug provocation | 21 |
| Gueant et al., 2015  (Validation study; Spain) | Amoxicillin (47.2),  Caphalosporin (33.2),  Other BL (19.6),  Penicillin (66.6) | Immediate only | Shock (78.5) | Age 16-60 and had history of immediate reaction to BL | No allergic diseases | 299/362 | -HLA-DRA (rs7192), OR=0.68 (P=0.003);  -HLA-DRA (rs8084), OR=0.68 (P=0.002);  -C5 (rs17612), OR=1.57 (P=0.053);  -ZNF300 (rs4958427), OR=0.81 (P=0.18) | - Skin test  - Total serum IgE level  - Drug provocation | - |
| Krebs et al., 2020  (Discovery study; Estonian Biobank, UK Biobank, Vanderbilt University Medical Center’s DNA Biobank (BioVU) | Penicillins and other BL | NA | Anaphylactic (2.4) | Self-reported penicillin allergy   - Identified by Z88.0 ICD10 code (UK Biobank)  - Targeted self-reported side effect of J01C* ATC drug group  (Beta-Lactam Antibacterial, Penicillins) from questionnaires (Estonian Biobank) - Clinical note extraction indicating “penicillin” in allergy section (BioVU) | Healthy control | 29,691/ 440,856 | -MHC (rs114892859), OR=1.47 (CI: 1.38,1.57), (P=1.29E-29)  - PTPN22 (rs2476601), OR=1.09 (CI: 1.06, 1.12), (P=2.68E-9)  -HLA-B*55:01, OR =1.41 (CI: 1.33,1.49), (P=2.04E-31) | NA | 17 |
| Krebs et al., 2020  (Validation study; 23andMe) | Penicillins | NA | NA | Self-reported questionnaires based on positive allergy test or allergic symptoms | Healthy control | 87,996/ 1,031,087 | -HLA-B*55:01, OR =1.30 (CI: 1.25, 1.34) (P=1E-47) | Allergy test (not specified) | - |
| Nicoletti et al., 2021  (Discovery study; EU) | **-Immediate:**  Penicillin (75), Cephalosporin (25)  **-Delayed:**  Penicillin (92), Cephalosporin (7.5),  Other BL (0.01) | 71.2/28.8 | **-Immediate:** angioedema (35), bronchospasm (24),  urticaria (34), hypertension (4)  **- Delayed:** AGEP(5),  DRESS (3), macular exanthem (79), SJS/TEN (13) | Specialist diagnosed BL allergy and positive functional validation | Healthy control | 930/ 9,217  Immediate (662), Delayed (268) | **Immediate:**  - HLA-II region (rs71542416), OR= 5.17 (CI: 3.40, 5.17), (P=1.2E-14)  - HLA-DRB1*10:01, OR= 2.95 (CI: 1.99, 4.36), (P=6E-8)  - HLA-DQA1*01:05, OR= 2.93 (CI: 1.92, 4.46), (P=5.4E-7)  - rs14632839, OR= 0.77 (CI: 0.67, 0.89), (P=0.0003)  **Delayed:**  HLA-DRB1*10:01, OR= 1.34 (CI: 0.55, 3.26), (P=0.5) | - specific IgE  - skin prick test  - Intradermal  - Oral provocation) | 17 |
| Nicoletti et al., 2021  (Validation study; EU) | Penicillin (100) | Immediate only | NA | Specialist diagnosed BL allergy and positive functional validation | Healthy control | 98/ 3,137 | - HLA-DRB1*10:01, OR= 2.80 (CI: 1.17, 6.71), (exact P=0.016) | - specific IgE  - skin prick test  - intradermal  - oral provocation) | - |
| Romano et al., 2021 (HLA typing by NGS; Italy) | **Delayed:**  Amoxicillin (20.8),  Ampicillin (12.5),  Bacampicillin (12.5),  Penicillin G (8.3),  Amoxicillin-Clavulanic acid (4.2),  Multiple drugs (41.7)  **Immediate:**  Multiple drugs (100)  (e.g. Amoxicillin, ampicillin, cephalosporin, penicillin G) | 45.4/54.5 | **Delayed:**  Multiple symptoms (70.8),  Maculopapular exanthema (20.8),  Edema (8.3)  **Immediate:**  Anaphylaxis (70.0)  Urticaria/angioedema (2.0)  Others (1.0) | Delayed reaction | Immediate reaction | 24/20 | HLA-DRB3*02:02:01:02;  HLA-DRB3*02:02:01:01 | - Skin test  - Patch test | 17 |
| Romano et al., 2021 (NGS, fine-mapping; Spain) | **Delayed:**  Amoxicillin (48.0)  Amoxicillin-Clavulanic acid (27.6)  Penicillin G (12.2)  Others (12.2)  **Immediate:**  Amoxicillin (54.9)  Amoxicillin-Clavulanic acid (33.9)  Penicillin G (4.8)  Cefuroxime (2.2)  Others (4.1) | 57.9/42.1 | **Delayed:**  Urticaria (71.4)  Urticaria/ angioedema (25.5)  Others (3.1)  **Immediate:**  Anaphylaxis (51.4)  Anaphylactic shock (15.8)  Urticaria (32.7) | Delayed reaction;  315, immediate reaction | Healthy control | 98 (Delayed), 315 (Immediate)/ 1,308 | **LD block findings**  **Delayed:**  HLA-DRB3 (rs9268835), OR=1.42 (CI: 1.05, 1.91);  HLA-DRB3 (rs6923504), OR=0.78 (CI: 0.57, 1.06)  HLA-DRB3 (rs6903608), OR=0.80 (CI: 0.59, 1.09)  HLA-DRB3 (rs9268838), OR=1.43 (CI: 1.06, 1.94)  **Immediate:**  HLA-DRB3 (rs9268835), OR=1.14 (CI: 0.94, 1.37);  HLA-DRB3 (rs6923504), OR=0.95 (CI: 0.79, 1.14)  HLA-DRB3 (rs6903608), OR=0.98 (CI: 0.81, 1.17)  HLA-DRB3 (rs9268838), OR=1.15 (CI: 0.95, 1.38) | - Skin test  - Patch test | - |

HLA=Human leukocyte antigen; NGS= Next generation sequencing; LD = Linkage disequilibrium

**Table S6** Quality assessment of eligible candidate-gene studies following STrengthening the REporting of Genetic Association Studies (STREGA) statement

|  | | Qiao, 2004 | Qiao, 2005 | Yang, 2005 | Guglielmi,  2006 | Guéant-  Rodriguez,  2006 | Qiao, 2007 | Apter, 2008 | Gao, 2008 | Guéant-  Rodriguez,  2008 | Huang, 2009 | Zhang, 2009  (china) | Zhang, 2010  (china) | Zhang, 2010  (china) | Ming, 2011 |
| --- | --- | --- | --- | --- | --- | --- | --- | --- | --- | --- | --- | --- | --- | --- | --- |
| Objectives | State if the study is the first report of a genetic association, a replication effort, or both. | Yes | Yes | Yes | Yes | Yes | Yes | Yes | Yes | Yes | Yes | Yes | Yes | Yes | Yes |
| **Methods** | |  |  |  |  |  |  |  |  |  |  |  |  |  |  |
| Participants | Give information on the criteria and methods for selection of subsets of participants from a larger study, when relevant. | Yes | Yes | Yes | Yes | Yes | Yes | Yes | Yes | Yes | Yes | Yes | Yes | Yes | Yes |
| Variables | Clearly define genetic exposures (genetic variants) using a widely used nomenclature system. | Yes | Yes | Yes | yes | Yes | Yes | Yes | Yes | Yes | Yes | yes | Yes | Yes | Yes |
|  | Identify variables likely to be associated with population stratification (confounding by ethnic origin). | No | No | No | No | No | No | Yes | No | No | No | No | No | No | No |
| Data sources Measurement | Describe laboratory methods, including source and storage of DNA, genotyping methods and platforms (including the allele-calling algorithm used, and its version), error rates, and call rates | Yes | Yes | Yes | Yes | Yes | Yes | Yes | Yes | Yes | Yes | yes | Yes | yes | Yes |
|  | State the laboratory/center where genotyping was done. | Yes | No | No | Yes | Yes | No | Yes | No | Yes | No | No | No | No | No |
|  | Describe comparability of laboratory methods if there is more than one group. | NA | NA | NA | NA | NA | NA | NA | NA | NA | NA | NA | NA | NA | NA |
|  | Specify whether genotypes were assigned using all of the data from the study simultaneously or in smaller batches. | yes | Yes | Yes | Yes | Yes | Yes | Yes | Yes | Yes | Yes | Yes | yes | Yes | Yes |
| Bias | For quantitative outcome variables, specify if any investigation of potential bias resulting from pharmacotherapy was undertaken. | No | No | No | No | No | No | No | No | No | No | No | No | No | No |
|  | If relevant, describe the nature and magnitude of the potential bias, and explain what approach was used to deal with this. | No | No | No | No | No | No | No | No | No | No | No | No | No | No |
| Study size | Explain how the study size was arrived at. | No | No | No | No | Yes | No | No | No | Yes | yes | No | No | No | No |
| Quantitative variables | If applicable, describe how effects of treatment were dealt with. | No | No | No | No | No | No | No | No | No | No | No | No | No | No |
| Statistical methods | State whether Hardy-Weinberg equilibrium was considered and, if so, how. | No | No | No | No | Yes | No | Yes | No | Yes | Yes | No | No | No | Yes |
|  | Describe any methods used for inferring genotypes or haplotypes. | No | No | No | No | No | Yes | Yes | No | No | Yes | No | No | No | Yes |
|  | Describe any methods used to assess or address population stratification | Yes | Yes | Yes | Yes | Yes | No | Yes | No | Yes | Yes | Yes | Yes | Yes | Yes |
|  | Describe any methods used to address multiple comparisons or to control risk of false-positive findings | NA | NA | NA | NA | No | No | Yes | No | No | No | No | No | No | No |
|  | Describe any methods used to address and correct for relatedness among subjects. | No | No | No | No | No | No | No | No | No | No | No | No | No | No |
| **Results** | |  |  |  |  |  |  |  |  |  |  |  |  |  |  |
| Participants | Report numbers of individuals in whom genotyping was attempted and numbers of individuals in whom genotyping was successful. | Yes | Yes | Yes | Yes | Yes | Yes | Yes | Yes | Yes | Yes | Yes | Yes | Yes | Yes |
| Descriptive data | Consider giving information by genotype. | Yes | Yes | Yes | Yes | Yes | Yes | Yes | Yes | Yes | Yes | Yes | Yes | Yes | Yes |
| Outcome | Report outcomes (phenotypes) for each genotype category over time. | NA | NA | NA | NA | NA | NA | NA | NA | NA | NA | NA | NA | NA | NA |
|  | Report numbers in each genotype category. | Yes | Yes | Yes | Yes | Yes | Yes | Yes | Yes | Yes | Yes | Yes | Yes | Yes | Yes |
|  | Report outcomes (phenotypes) for each genotype category. | NA | NA | NA | NA | NA | NA | NA | NA | NA | NA | NA | NA | NA | NA |
| Main results | Report results of any adjustments for multiple comparisons. | NA | NA | NA | NA | NA | NA | Yes | NA | NA | NA | NA | NA | NA | NA |
| Other analysis | If numerous genetic exposures (genetic variants) were examined, summarize results from all analyses undertaken. | NA | NA | Yes | Yes | Yes | Yes | Yes | NA | NA | Yes | Yes | Yes | Yes | Yes |
|  | If detailed results are available elsewhere, state how they can be accessed. | NA | NA | NA | NA | No | NA | NA | NA | NA | NA | NA | NA | NA | NA |
| **SCORE** | Total number of ‘Yes’ / number of items | 10/25 | 9/25 | 10/25 | 11/25 | 13/25 | 10/25 | 16/25 | 8/25 | 12/25 | 13/25 | 10/25 | 10/25 | 10/25 | 12/25 |

**Table S6** Quality assessment of eligible candidate-gene studies (continued)

|  | | Chen, 2012 | Cornejo-  Garcia, 2012 | Huang, 2012 | Nam, 2012 | Bursztejn, 2013 | Li, 2015 | Cornejo-  Garcia, 2016 | Khadhim, 2018 | Amo, 2019 | Ksouri, 2019 | Rivera-Reigada, 2020 | Yang, 2006 | Singvicharn, 2021 |
| --- | --- | --- | --- | --- | --- | --- | --- | --- | --- | --- | --- | --- | --- | --- |
| Objectives | State if the study is the first report of a genetic association, a replication effort, or both. | Yes | Yes | Yes | Yes | Yes | Yes | yes | Yes | Yes | Yes | Yes | Yes | Yes |
| **Methods** | |  |  |  |  |  |  |  |  |  |  |  |  |  |
| Participants | Give information on the criteria and methods for selection of subsets of participants from a larger study, when relevant. | Yes | Yes | Yes | Yes | Yes | Yes | Yes | Yes | Yes | Yes | Yes | Yes | Yes |
| Variables | Clearly define genetic exposures (genetic variants) using a widely used nomenclature system. | Yes | Yes | Yes | Yes | Yes | Yes | Yes | Yes | Yes | Yes | Yes | Yes | Yes |
|  | Identify variables likely to be associated with population stratification (confounding by ethnic origin). | No | No | No | No | No | No | No | No | No | No | No | No | Yes |
| Data sources Measurement | Describe laboratory methods, including source and storage of DNA, genotyping methods and platforms (including the allele-calling algorithm used, and its version), error rates, and call rates | yes | No | yes | yes | Yes | Yes | yes | Yes | Yes | Yes | No | Yes | Yes |
|  | State the laboratory/center where genotyping was done. | Yes | Yes | No | Yes | Yes | Yes | Yes | Yes | Yes | Yes | No | Yes | Yes |
|  | Describe comparability of laboratory methods if there is more than one group. | NA | NA | NA | NA | No | NA | NA | NA | NA | NA | NA | NA | NA |
|  | Specify whether genotypes were assigned using all of the data from the study simultaneously or in smaller batches. | Yes | Yes | Yes | Yes | yes | Yes | Yes | Yes | Yes | Yes | Yes | No | Yes |
| Bias | For quantitative outcome variables, specify if any investigation of potential bias resulting from pharmacotherapy was undertaken. | No | No | No | No | No | No | No | No | No | No | No | No | No |
|  | If relevant, describe the nature and magnitude of the potential bias, and explain what approach was used to deal with this. | No | No | No | No | No | No | No | No | No | No | Yes | No | No |
| Study size | Explain how the study size was arrived at. | No | yes | No | No | yes | No | No | No | Yes | No | No | No | No |
| Quantitative variables | If applicable, describe how effects of treatment were dealt with. | No | No | No | yes | No | No | No | No | Yes | No | No | No | No |
| Statistical methods | State whether Hardy-Weinberg equilibrium was considered and, if so, how. | Yes | No | Yes | No | No | Yes | Yes | Yes | Yes | Yes | No | No | No |
|  | Describe any methods used for inferring genotypes or haplotypes. | Yes | No | No | No | No | Yes | No | No | No | Yes | No | Yes | Yes |
|  | Describe any methods used to assess or address population stratification | Yes | No | Yes | No | Yes | Yes | Yes | Yes | No | No | No | Yes | Yes |
|  | Describe any methods used to address multiple comparisons or to control risk of false-positive findings | Yes | Yes | No | Yes | No | No | Yes | No | Yes | No | No | No | Yes |
|  | Describe any methods used to address and correct for relatedness among subjects. | No | No | No | No | No | No | No | No | No | No | No | No | No |
| **Results** | |  |  |  |  |  |  |  |  |  |  |  |  |  |
| Participants | Report numbers of individuals in whom genotyping was attempted and numbers of individuals in whom genotyping was successful. | Yes | Yes | Yes | Yes | Yes | Yes | Yes | Yes | Yes | Yes | Yes | Yes | Yes |
| Descriptive data | Consider giving information by genotype. | Yes | Yes | Yes | Yes | yes | Yes | Yes | Yes | Yes | Yes | Yes | Yes | Yes |
| Outcome | Report outcomes (phenotypes) for each genotype category over time. | NA | NA | NA | NA | NA | NA | NA | NA | NA | NA | NA | NA | NA |
|  | Report numbers in each genotype category. | Yes | Yes | Yes | Yes | Yes | Yes | Yes | Yes | Yes | Yes | Yes | Yes | Yes |
|  | Report outcomes (phenotypes) for each genotype category. | NA | NA | NA | NA | NA | NA | NA | NA | NA | NA | NA | NA | NA |
| Main results | Report results of any adjustments for multiple comparisons. | NA | NA | NA | Yes | No | No | Yes | No | yes | No | NA | NA | Yes |
| Other analysis | If numerous genetic exposures (genetic variants) were examined, summarize results from all analyses undertaken. | Yes | Yes | No | Yes | Yes | Yes | Yes | Yes | Yes | Yes | Yes | Yes | Yes |
|  | If detailed results are available elsewhere, state how they can be accessed. | NA | NA | NA | No | No | No | No | No | Yes | No | Yes | NA | NA |
| **SCORE** | Total number of ‘Yes’ / number of items | 14/25 | 11/25 | 10/25 | 13/25 | 12/25 | 13/25 | 14/25 | 12/25 | 16/25 | 12/25 | 10/25 | 11/25 | 15/25 |

**Table S6** Quality assessment of eligible GWAS following STrengthening the REporting of Genetic Association Studies (STREGA) statement

|  | | Gueant, 2015 | Krebs, 2020 | Nicoletti, 2021 | Romano, 2022 |
| --- | --- | --- | --- | --- | --- |
| Objectives | State if the study is the first report of a genetic association, a replication effort, or both. | Yes | Yes | Yes | Yes |
| **Methods** | |  |  |  |  |
| Participants | Give information on the criteria and methods for selection of subsets of participants from a larger study, when relevant. | Yes | Yes | Yes | Yes |
| Variables | Clearly define genetic exposures (genetic variants) using a widely used nomenclature system. | Yes | Yes | Yes | Yes |
|  | Identify variables likely to be associated with population stratification (confounding by ethnic origin). | Yes | No | Yes | No |
| Data sources Measurement | Describe laboratory methods, including source and storage of DNA, genotyping methods and platforms (including the allele-calling algorithm used, and its version), error rates, and call rates | Yes | Yes | Yes | Yes |
|  | State the laboratory/center where genotyping was done. | Yes | Yes | Yes | Yes |
|  | Describe comparability of laboratory methods if there is more than one group. | Yes | Yes | Yes | No |
|  | Specify whether genotypes were assigned using all of the data from the study simultaneously or in smaller batches. | Yes | Yes | Yes | Yes |
| Bias | For quantitative outcome variables, specify if any investigation of potential bias resulting from pharmacotherapy was undertaken. | No | No | No | No |
|  | If relevant, describe the nature and magnitude of the potential bias, and explain what approach was used to deal with this. | Yes | Yes | Yes | Yes |
| Study size | Explain how the study size was arrived at. | Yes | No | No | No |
| Quantitative variables | If applicable, describe how effects of treatment were dealt with. | No | No | No | Yes |
| Statistical methods | State whether Hardy-Weinberg equilibrium was considered and, if so, how. | Yes | Yes | Yes | Yes |
|  | Describe any methods used for inferring genotypes or haplotypes. | Yes | Yes | Yes | Yes |
|  | Describe any methods used to assess or address population stratification | Yes | Yes | Yes | Yes |
|  | Describe any methods used to address multiple comparisons or to control risk of false-positive findings | Yes | Yes | Yes | No |
|  | Describe any methods used to address and correct for relatedness among subjects. | Yes | Yes | No | Yes |
| **Results** | |  |  |  |  |
| Participants | Report numbers of individuals in whom genotyping was attempted and numbers of individuals in whom genotyping was successful. | Yes | Yes | Yes | Yes |
| Descriptive data | Consider giving information by genotype. | Yes | No | NA | Yes |
| Outcome | Report outcomes (phenotypes) for each genotype category over time. | NA | NA | NA | NA |
|  | Report numbers in each genotype category. | Yes | No | No | Yes |
|  | Report outcomes (phenotypes) for each genotype category. | NA | NA | NA | NA |
| Main results | Report results of any adjustments for multiple comparisons. | Yes | Yes | Yes | No |
| Other analysis | If numerous genetic exposures (genetic variants) were examined, summarize results from all analyses undertaken. | Yes | Yes | Yes | Yes |
|  | If detailed results are available elsewhere, state how they can be accessed. | Yes | Yes | Yes | Yes |
| **SCORE** | Total number of ‘Yes’ / number of items | 21/25 | 17/25 | 17/25 | 17/25 |

**Table S6.1** Genotypic and allelic effect of ***IL-4R*: rs1801275 (Q576R)** in patients with Beta-lactams (BL) allergy

| **Author, Year** | **Ethnicity** | **HWE P-value** | **Case genotype** | | | **Control genotype** | | | **Case MAF** | **Control MAF** | **Odd ratio (95% CI)** | | | | | |
| --- | --- | --- | --- | --- | --- | --- | --- | --- | --- | --- | --- | --- | --- | --- | --- | --- |
|  |  |  | **AA** | **AG** | **GG** | **AA** | **AG** | **GG** |  |  | **G vs A** | **GG vs AA** | **GA vs AA** | **GG vs AA+AG** | **GG+AG vs AA** | **GG+AA vs AG** |
| Qiao et al., 2005 | Chinese | 0.4703 | 120 | 31 | 5 | 52 | 32 | 3 | 0.13 | 0.22 | 0.54  (0.33, 0.88) | 0.72  (0.17, 3.13) | 0.42  (0.23, 0.76) | 0.93  (0.22, 3.98) | 0.45  (0.25, 0.79) | 2.35  (1.30,4.22) |
| Gouglemi et al., 2006 | Caucasian | 0.0827 | 29 | 15 | 0 | 33 | 7 | 2 | 0.17 | 0.13 | 1.36  (0.59, 3.17) | 0.23  (0.01, 4.92) | 2.44  (0.87, 6.81) | 0.18  (0.01, 3.91) | 1.90  (0.72, 4.98) | 0.39  (0.14,1.08) |
| Gueant-Rodriguez et al., 2006 | Italian | 0.3324 | 162 | 41 | 2 | 182 | 77 | 5 | 0.11 | 0.16 | 0.62  (0.42, 0.92) | 0.45  (0.09, 2.35) | 0.60  (0.39, 0.92) | 0.51  (0.10, 2.66) | 0.59  (0.38, 0.90) | 1.65  (1.07,2.54) |
| Huang et al., 2009 | Chinese | 0.6131 | 185 | 52 | 5 | 142 | 68 | 10 | 0.13 | 0.20 | 0.59  (0.41, 0.84) | 0.38  (0.13, 1.15) | 0.59  (0.38, 0.90) | 0.44  (0.15, 1.32) | 0.56  (0.37, 0.84) | 1.63  (1.07,2.49) |
| Zhang et al., 2010 | Chinese | 0.2733 | 40 | 19 | 1 | 32 | 10 | 2 | 0.18 | 0.16 | 1.12  (0.53, 2.35) | 0.40  (0.03, 4.61) | 1.52  (0.62, 3.72) | 0.36  (0.03, 4.05) | 1.33  (0.57, 3.13) | 0.63  (0.26,1.55) |
| Cornejo-Garcia et al., 2012 | Spanish | 0.0015 | 228 | 94 | 18 | 171 | 122 | 47 | 0.19 | 0.32 | NA | NA | NA | NA | NA | NA |
| Li et al., 2015 | Chinese | 0.2733 | 40 | 19 | 4 | 20 | 10 | 0 | 0.21 | 0.17 | 1.36  (0.61, 3.04) | 4.56  (0.23,88.77) | 0.95  (0.37, 2.42) | 4.61  (0.24,88.51) | 1.15  (0.46, 2.87) | 1.16  (0.46,2.94) |
| Khadhim et al., 2018 | Iraq | 0.5557 | 28 | 12 | 10 | 19 | 22 | 9 | 0.32 | 0.40 | 0.71  (0.40, 1.26) | 0.75  (0.26, 2.20) | 0.37  (0.15, 0.92) | 1.14  (0.42, 3.10) | 0.48  (0.22, 1.07) | 2.49  (1.06,5.86) |
| **Pooled** | | | | | | | | | | | **0.73**  **(0.57, 0.93)** | 0.59  (0.34, 1.04) | 0.71  (0.49,1.06) | 0.73  (0.43, 1.25) | **0.70**  **(0.50, 0.99)** | 1.38  (0.93,2.05) |

**Table S6.2** Genotypic and allelic effect of ***IL-13*: rs20541: G>A (R130Q)** in patients with Beta-lactams (BL) allergy

| **Author, Year** | **Ethnicity** | **HWE P-value** | **Case genotype** | | | **Control genotype** | | | **Case MAF** | **Control MAF** | **Odd ratio (95% CI)** | | | | | |
| --- | --- | --- | --- | --- | --- | --- | --- | --- | --- | --- | --- | --- | --- | --- | --- | --- |
|  |  |  | **GG** | **GA** | **AA** | **GG** | **GA** | **AA** |  |  | **A vs G** | **AA vs GG** | **GA vs GG** | **AA vs GG+GA** | **AA+GA vs GG** | **AA+GG vs GA** |
| Yang et al., 2005 | Chinese | 0.7030 | 72 | 72 | 11 | 39 | 36 | 10 | 0.30 | 0.33 | 0.89  (0.59, 1.32) | 0.60  (0.23,1.53) | 1.08  (0.62, 1.89) | 0.57  (0.23, 1.41) | 0.98  (0.57, 1.66) | 0.85  (0.50,1.44) |
| Gouglemi et al., 2006 | French | 0.6451 | 24 | 18 | 2 | 29 | 14 | 1 | 0.25 | 0.18 | 1.50  (0.73, 3.10) | 2.42  (0.21,28.30) | 1.55  (0.64, 3.76) | 2.05  (0.18,23.44) | 1.61  (0.68, 3.81) | 0.67  (0.28,1.62) |
| Gueant-Rodriquez et al., 2006 | Italian | 0.1290 | 120 | 85 | 4 | 179 | 73 | 13 | 0.22 | 0.19 | 1.25  (0.91, 1.71) | 0.46  (0.15, 1.44) | 1.74  (1.18, 2.56) | 0.38  (0.12, 1.18) | 1.54  (1.06, 2.25) | 0.55  (0.38,0.82) |
| Cornejo-Garcia et al., 2012 | Spanish | 0.3198 | 229 | 94 | 17 | 244 | 86 | 11 | 0.19 | 0.16 | 1.23  (0.93, 1.63) | 1.65  (0.76, 3.59) | 1.16  (0.83, 1.64) | 1.58  (0.73, 3.42) | 1.22  (0.88, 1.69) | 0.88  (0.63,1.24) |
| Pooled | | | | | | | | | | | 1.17  (0.98, 1.40) | 0.90  (0.43, 1.86) | **1.34**  **(1.07, 1.68)** | 0.82  (0.38, 1.78) | **1.29**  **(1.04, 1.60)** | **0.74**  **(0.59,0.92)** |

**Table S6.3** Genotypic and allelic effect of ***IL-13*: rs20541: C>T** in patients with Beta-lactams (BL) allergy

| **Author, Year** | **Ethnicity** | **HWE P-value** | **Case genotype** | | | **Control genotype** | | | **Case MAF** | **Control MAF** | **Odd ratio (95% CI)** | | | | | |
| --- | --- | --- | --- | --- | --- | --- | --- | --- | --- | --- | --- | --- | --- | --- | --- | --- |
|  |  |  | **CC** | **CT** | **TT** | **CC** | **CT** | **TT** |  |  | **T vs C** | **TT vs CC** | **CT vs CC** | **TT vs CC+CT** | **TT+CT vs CC** | **TT+CC vs CT** |
| Li et al., 2015 | Chinese | 0.2190 | 28 | 31 | 4 | 13 | 11 | 6 | 0.31 | 0.38 | 0.72  (0.36,1.45) | 0.31  (0.06,1.60) | 1.31  (0.46,3.80) | 0.27  (0.05,1.28) | 0.96  (0.36,2.50) | 0.60  (0.22,1.59) |
| Ksouri et al., 2019 | Algeria | <0.0001 | 152 | 42 | 5 | 87 | 12 | 7 | 0.13 | 0.12 | NA | NA | NA | NA | NA | NA |
| Pooled | | | | | | | | | | | NA | NA | NA | NA | NA | NA |

**Table S6.4** Genotypic and allelic effect of ***FceRIb* : rs569108; A>G (E237G)** in patients with Beta-lactams (BL) allergy

| **Author, Year** | **Ethnicity** | **HWE P-value** | **Case genotype** | | | **Control genotype** | | | **Case MAF** | **Control MAF** | **Odd ratio (95% CI)** | | | | | |
| --- | --- | --- | --- | --- | --- | --- | --- | --- | --- | --- | --- | --- | --- | --- | --- | --- |
|  |  |  | **AA** | **AG** | **GG** | **AA** | **AG** | **GG** |  |  | **G vs A** | **GG vs AA** | **GA vs AA** | **GG vs AA+AG** | **GG+AG vs AA** | **GG+AA vs AG** |
| Qiao et al., 2004 | Chinese | 0.1561 | 106 | 47 | 1 | 63 | 23 | 5 | 0.18 | 0.14 | 0.85  (0.53, 1.39) | 0.12  (0.01, 1.04) | 1.21  (0.67, 2.19) | 0.11  (0.01, 0.98) | 1.02  (0.58, 1.79) | 0.77  (0.43,1.38) |
| Gouglemi et al., 2006 | French | 0.6895 | 40 | 4 | 0 | 39 | 5 | 0 | 0.05 | 0.06 | 0.79  (0.21, 3.05) | 0.98  (0.02,50.37) | 0.78  (0.19, 3.12) | 1.00  (0.02,51.52) | 0.78  (0.19, 3.12) | 1.28  (0.32,5.13) |
| Zhang et al., 2010 | Chinese | 0.5117 | 33 | 17 | 1 | 35 | 17 | 1 | 0.19 | 0.18 | 1.05  (0.52, 2.12) | 1.06  (0.06,17.66) | 1.06  (0.47, 2.42) | 1.04  (0.06,17.08) | 1.06  (0.47, 2.38) | 0.94  (0.42,2.14) |
| Pooled | | | | | | | | | | | 0.90  (0.61, 1.32) | 0.29  (0.07, 1.24) | 1.11  (0.71, 1.75) | 0.29  (0.07, 1.19) | 1.00  (0.65, 1.55) | 0.86  (0.55,1.35) |

**Table S6.5** Genotypic and allelic effect of ***IL-4R* : rs1805010 (I50V)** in patients with Beta-lactams (BL) allergy

| **Author, Year** | **Ethnicity** | **HWE P-value** | **Case genotype** | | | **Control genotype** | | | **Case MAF** | **Control MAF** | **Odd ratio (95% CI)** | | | | | |
| --- | --- | --- | --- | --- | --- | --- | --- | --- | --- | --- | --- | --- | --- | --- | --- | --- |
|  |  |  | **AA** | **AG** | **GG** | **AA** | **AG** | **GG** |  |  | **G vs A** | **GG vs AA** | **GA vs AA** | **GG vs AA+AG** | **GG+AG vs AA** | **GG+AA vs AG** |
| Gouglemi et al., 2006 | French | 0.9800 | 7 | 26 | 11 | 7 | 21 | 16 | 0.55 | 0.60 | 0.79  (0.44,1.44) | 0.69  (0.19, 2,52) | 1.24  (0.37, 4.09) | 0.58  (0.23, 1.46) | 1.00  (0.32, 3.13) | 0.63  (0.27,1.47) |
| Gueant-Rodriquez et al., 2006 | Italian | 0.0722 | 72 | 90 | 48 | 55 | 147 | 63 | 0.44 | 0.52 | 0.75  (0.58,0.97) | 0.58  (0.35, 0.97) | 0.47  (0.30, 0.72) | 0.95  (0.62, 1.46) | 0.50  (0.33, 0.76) | 1.66  (1.15,2.39) |
| Apter et al., 2008 | USA | 0.6335 | 5 | 12 | 4 | 11 | 17 | 9 | 0.48 | 0.47 | 1.01  (0.47,2.16) | 0.98  (0.20, 4.76) | 1.55  (0.43, 5.64) | 0.73  (0.19, 2.75) | 1.35  (0.40, 4.62) | 0.64  (0.22,1.88) |
| Huang et al., 2009 | Chinese | 0.1138 | 58 | 128 | 56 | 41 | 121 | 58 | 0.50 | 0.54 | 0.84  (0.65,1.09) | 0.68  (0.40, 1.17) | 0.75  (0.47, 1.20) | 0.84  (0.55, 1.28) | 0.73  (0.46, 1.14) | 1.09  (0.75,1.57) |
| Cornejo-Garcia et al., 2012 | Spanish | 0.1316 | 108 | 166 | 66 | 165 | 135 | 40 | 0.44 | 0.32 | 1.69  (1.35,2.11) | 2.52  (1.59, 4.00) | 1.88  (1.35, 2.62) | 1.81  (1.18, 2.76) | 2.03  (1.48, 2.77) | 0.69  (0.51,0.94) |
| Ksouri et al., 2019 | Algeria | 0.8948 | 44 | 86 | 69 | 32 | 48 | 19 | 0.56 | 0.43 | 1.68  (1.19,2.36) | 2.64  (1.34, 5.22) | 1.30  (0.73, 2.32) | 2.23  (1.25, 3.99) | 1.68  (0.98, 2.88) | 1.24  (0.76,2.01) |
| Pooled | | | | | | | | | | | 1.08  (0.76,1.54) | 1.14  (0.59, 2.23) | 1.04  (0.60, 1.81) | 1.15  (0.77, 1.72) | 1.08  (0.61, 1.92) | 1.00  (0.70,1.41) |

**Table S6.6** Genotypic and allelic effect of ***IL-4Ra*: rs1805015 (S478P)** in patients with Beta-lactams (BL) allergy

| **Author, Year** | **Ethnicity** | **HWE P-value** | **Case genotype** | | | **Control genotype** | | | **Case MAF** | **Control MAF** | **Odd ratio (95% CI)** | | | | | |
| --- | --- | --- | --- | --- | --- | --- | --- | --- | --- | --- | --- | --- | --- | --- | --- | --- |
|  |  |  | **TT** | **TC** | **CC** | **TT** | **TC** | **CC** |  |  | **C vs T** | **CC vs TT** | **TC vs TT** | **CC vs TT+TC** | **CC+TC vs TT** | **CC+TT vs TC** |
| Gueant-Rodriquez et al., 2006 | Italian | 0.6412 | 168 | 39 | 3 | 197 | 64 | 4 | 0.11 | 0.14 | 0.76  (0.51, 1.13) | 0.88  (0.19, 3.99) | 0.71  (0.46,1.12) | 0.95  (0.21, 4.27) | 0.72  (0.47,1.12) | 1.40  (0.89,2.18) |
| Cornejo-Garcia et al., 2012 | Spanish | 0.6591 | 239 | 91 | 10 | 240 | 90 | 10 | 0.16 | 0.16 | 1.01  (0.76, 1.35) | 1.00  (0.41, 2.46) | 1.02  (0.72,1.43) | 1.00  (0.41, 2.43) | 1.01  (0.73, 1.41) | 0.99  (0.70,1.38) |
| Ksouri et al., 2019 | Algeria | 0.1505 | 139 | 54 | 6 | 74 | 25 | 0 | 0.17 | 0.13 | 1.38  (0.84, 2.26) | 6.94  (0.39,124.94) | 1.15  (0.66,2.00) | 6.68  (0.37,119.87) | 1.28  (0.74, 2.20) | 0.91  (0.52,1.57) |
| Pooled | | | | | | | | | | | 0.99  (0.75, 1.32) | 1.24  (0.61, 2.53) | 0.94  (0.73,1.19) | 1.25  (0.62, 2.54) | 0.96  (0.72, 1.28) | 1.08  (0.84,1.37) |

**Table S6.7** Genotypic and allelic effect of ***IL-13*: rs1800925 (-1112C/T)** in patients with Beta-lactams (BL) allergy

| **Author, Year** | **Ethnicity** | **HWE P-value** | **Case genotype** | | | **Control genotype** | | | **Case MAF** | **Control MAF** | **Odd ratio (95% CI)** | | | | | |
| --- | --- | --- | --- | --- | --- | --- | --- | --- | --- | --- | --- | --- | --- | --- | --- | --- |
|  |  |  | **CC** | **CT** | **TT** | **CC** | **CT** | **TT** |  |  | **T vs C** | **TT vs CC** | **CT vs CC** | **TT vs CC+CT** | **TT+CT vs CC** | **TT+CC vs CT** |
| Gouglemi et al., 2006 | French | 0.7585 | 22 | 19 | 3 | 28 | 13 | 2 | 0.28 | 0.20 | 1.61  (0.80, 3,26) | 1.91  (0.29, 12.44) | 1.86  (0.76,4.57) | 1.50  (0.24, 9.45) | 1.87  (0.79, 4.42) | 0.57  (0.24,1.38) |
| Gueant-Rodriquez et al., 2006 | Italian | 0.4051 | 88 | 61 | 1 | 176 | 71 | 10 | 0.21 | 0.18 | 1.24  (0.86, 1.77) | 0.20  (0.03, 1.59) | 1.72  (1.12,2.63) | 0.17  (0.02, 1.31) | 1.53  (1.01, 2.33) | 0.56  (0.36,0.85) |
| Li et al., 2015 | Chinese | 0.9031 | 46 | 16 | 1 | 17 | 11 | 2 | 0.14 | 0.25 | 0.50  (0.23, 1.08) | 0.18  (0.02, 2.17) | 0.54  (0.21,1.39) | 0.23  (0.02, 2.59) | 0.48  (0.19, 1.20) | 1.70  (0.67,4.33) |
| Pooled | | | | | | | | | | | 1.05  (0.59, 1.86) | 0.46  (0.10, 2.26) | 1.29  (0.65,2.55) | 0.43  (0.10, 1.82) | 1.18  (0.58, 2.38) | 0.75  (0.39,1.44) |

**Table S6.8** Genotypic and allelic effect of ***IL-4*: rs2070874 (-33C/T)** in patients with Beta-lactams (BL) allergy

| **Author, Year** | **Ethnicity** | **HWE P-value** | **Case genotype** | | | **Control genotype** | | | **Case MAF** | **Control MAF** | **Odd ratio (95% CI)** | | | | | |
| --- | --- | --- | --- | --- | --- | --- | --- | --- | --- | --- | --- | --- | --- | --- | --- | --- |
|  |  |  | **CC** | **CT** | **TT** | **CC** | **CT** | **TT** |  |  | **T vs C** | **TT vs CC** | **CT vs CC** | **TT vs CC+CT** | **TT+CT vs CC** | **TT+CC vs CT** |
| Apter et al., 2008 | USA | 0.0239 | 11 | 10 | 1 | 30 | 7 | 3 | 0.27 | 0.16 | NA | NA | NA | NA | NA | NA |
| Cornejo-Garcia et al., 2012 | Spanish | 0.0945 | 238 | 97 | 5 | 257 | 73 | 10 | 0.16 | 0.14 | 1.18  (0.87, 1.59) | 0.54  (0.18, 1.60) | 1.43  (1.01,2.04) | 0.49  (0.17,1.46) | 1.33  (0.95,1.86) | 0.68  (0.48,0.97) |
| Rivera-Reigada et al., 2020 | Spanish | 0.9805 | 16 | 2 | 0 | 54 | 21 | 2 | 0.05 | 0.16 | 0.30  (0.07, 1.35) | 0.66  (0.03, 14.46) | 0.32  (0.07,1.52) | 0.82  (0.04,17.74) | 0.29  (0.06,1.38) | 3.00  (0.63,14.18) |
| Pooled | | | | | | | | | | | 0.73  (0.21,2.61) | 0.55  (0.20, 1.55) | 0.83  (0.20,3.41) | 0.52  (0.19,1.45) | 0.76  (0.18,3.17) | 1.17  (0.29,4.71) |

**Table S6.9** Genotypic and allelic effect of ***IL-4*: -589C/T** in patients with Beta-lactams (BL) allergy

| **Author, Year** | **Ethnicity** | **HWE P-value** | **Case genotype** | | | **Control genotype** | | | **Case MAF** | **Control MAF** | **Odd ratio (95% CI)** | | | | | |
| --- | --- | --- | --- | --- | --- | --- | --- | --- | --- | --- | --- | --- | --- | --- | --- | --- |
|  |  |  | **CC** | **CT** | **TT** | **CC** | **CT** | **TT** |  |  | **T vs C** | **TT vs CC** | **CT vs CC** | **TT vs CC+CT** | **TT+CT vs CC** | **TT+CC vs CT** |
| Gouglemi et al., 2006 | French | 0.2949 | 31 | 12 | 1 | 32 | 12 | 0 | 0.16 | 0.14 | 1.20  (0.52,2.76) | 3.10  (0.12,78.87) | 1.03  (0.40,2.64) | 3.07  (0.12,77.41) | 1.12  (0.44,2.83) | 1.00  (0.39,2.56) |
| Zhang et al., 2009 | Chinese | 0.4583 | 0 | 17 | 33 | 3 | 24 | 28 | 0.83 | 0.73 | 1.83  (0.94,3.58) | 8.23  (0.41,166.09) | 5.00  (0.24,103.07) | 1.87  (0.85,4.12) | 6.73  (0.34,133.66) | 1.50  (0.68,3.32) |
| Pooled | | | | | | | | | | | 1.55  (0.92,2.61) | 5.23  (0.63,48.35) | 1.26  (0.53,3.02) | 1.93  (0.90,4.15) | 1.43  (0.61,3.35) | 1.27  (0.69,2.32) |

**Table S6.10** Genotypic and allelic effect of ***IL-10*: rs1800871** in patients with Beta-lactams (BL) allergy

| **Author, Year** | **Ethnicity** | **HWE P-value** | **Case genotype** | | | **Control genotype** | | | **Case MAF** | **Control MAF** | **Odd ratio (95% CI)** | | | | | |
| --- | --- | --- | --- | --- | --- | --- | --- | --- | --- | --- | --- | --- | --- | --- | --- | --- |
|  |  |  | **TT** | **TC** | **CC** | **TT** | **TC** | **CC** |  |  | **C vs T** | **CC vs TT** | **TC vs TT** | **CC vs TT+TC** | **CC+TC vs TT** | **CC+TT vs TC** |
| Gouglemi et al., 2006 | French | 0.2828 | 22 | 16 | 6 | 24 | 15 | 5 | 0.32 | 0.28 | 1.18  (0.62, 2.24) | 1.31  (0.35, 4.90) | 1.16  (0.47,2.90) | 1.23  (0.35, 4.38) | 1.20  (0.52, 2.77) | 0.91  (0.38,2.17) |
| Qiao et al., 2007 | Chinese | 0.1615 | 61 | 35 | 6 | 48 | 29 | 9 | 0.23 | 0.27 | 0.80  (0.50, 1.27) | 0.52  (0.17, 1.58) | 0.95  (0.51,1.77) | 0.53  (0.18, 1.57) | 0.85  (0.47,1.52) | 0.97  (0.53,1.78) |
| Apter et al., 2008 | USA | 0.0006 | 12 | 8 | 2 | 22 | 6 | 7 | 0.27 | 0.27 | NA | NA | NA | NA | NA | NA |
| Li et al., 2015 | Chinese | 0.1221 | 26 | 27 | 11 | 14 | 10 | 6 | 0.42 | 0.37 | 1.07  (0.57, 2.02) | 0.99  (0.30, 3.24) | 1.45  (0.55,3.85) | 0.83  (0.27,2.51) | 1.28  (0.53,3.06) | 0.69  (0.28,1.70) |
| Pooled | | | | | | | | | | | 0.95  (0.69, 1.32) | 0.83  (0.42, 1.64) | 1.10  (0.70,1.72) | 0.78  (0.41, 1.50) | 1.02  (0.67, 1.55) | 0.88  (0.57,1.36) |

**Table S6.11** Genotypic and allelic effect of ***IL-10*: rs1800872** **: (-592C-A)** in patients with Beta-lactams (BL) allergy

| **Author, Year** | **Ethnicity** | **HWE P-value** | **Case genotype** | | | **Control genotype** | | | **Case MAF** | **Control MAF** | **Odd ratio (95% CI)** | | | | | |
| --- | --- | --- | --- | --- | --- | --- | --- | --- | --- | --- | --- | --- | --- | --- | --- | --- |
|  |  |  | **CC** | **CA** | **AA** | **CC** | **CA** | **AA** |  |  | **A vs C** | **AA vs CC** | **CA vs CC** | **AA vs CC+CA** | **AA+CA vs CC** | **AA+CC vs CA** |
| Gouglemi et al., 2006 | French | 0.2828 | 22 | 16 | 6 | 24 | 15 | 5 | 0.32 | 0.28 | 1.18  (0.62, 2.24) | 1.31  (0.35, 4.90) | 1.16  (0.47,2.90) | 1.23  (0.35, 4.38) | 1.20  (0.52, 2.77) | 0.91  (0.38,2.17) |
| Li et al., 2015 | Chinese | 0.1221 | 11 | 26 | 26 | 6 | 10 | 14 | 0.63 | 0.63 | 0.94  (0.50, 1.78) | 1.01  (0.31, 3.32) | 1.42  (0.41,4.87) | 0.80  (0.33, 1.93) | 1.18  (0.39, 3.57) | 0.71  (0.29,1.77) |
| Pooled | | | | | | | | | | | 1.05  (0.67, 1.65) | 1.14  (0.47, 2.74) | 1.25  (0.60,2.60) | 0.92  (0.45, 1.89) | 1.19  (0.61, 2.33) | 0.81  (0.43,1.51) |

**Table S6.12** Genotypic and allelic effect of ***IL-10*: rs1800896** **: (-1082G/A)** in patients with Beta-lactams (BL) allergy

| **Author, Year** | **Ethnicity** | **HWE P-value** | **Case genotype** | | | **Control genotype** | | | **Case MAF** | **Control MAF** | **Odd ratio (95% CI)** | | | | | |
| --- | --- | --- | --- | --- | --- | --- | --- | --- | --- | --- | --- | --- | --- | --- | --- | --- |
|  |  |  | **AA** | **AG** | **GG** | **AA** | **AG** | **GG** |  |  | **G vs A** | **GG vs AA** | **GA vs AA** | **GG vs AA+AG** | **GG+AG vs AA** | **GG+AA vs AG** |
| Gouglemi et al., 2006 | French | 0.6948 | 14 | 20 | 10 | 19 | 17 | 5 | 0.45 | 0.33 | 1.70  (0.91,3.17) | 2.71  (0.76, 9.73) | 1.60  (0.62,4.11) | 2.12  (0.66, 6.83) | 1.85  (0.77, 4.47) | 0.85  (0.36,2.01) |
| Qiao et al., 2007 | Chinese | 0.1085 | 51 | 39 | 12 | 35 | 34 | 17 | 0.31 | 0.40 | 0.68  (0.45,1.05) | 0.48  (0.21, 1.14) | 0.79  (0.42,1.48) | 0.54  (0.24, 1.21) | 0.69  (0.38,1.22) | 1.06  (0.59,1.90) |
| Li et al., 2015 | Chinese | 0.6956 | 57 | 7 | 2 | 26 | 4 | 0 | 0.08 | 0.07 | 1.27  (0.39, 4.17) | 2.30  (0.11,49.96) | 0.80  (0.21,2.97) | 2.36  (0.11,50.77) | 1.03  (0.29, 3.64) | 1.30  (0.35,4.82) |
| Pooled | | | | | | | | | | | 1.08  (0.55, 2.11) | 1.18  (0.30, 4.61) | 0.95  (0.59,1.55) | 1.07  (0.36, 3.20) | 1.02  (0.53, 1.95) | 1.02  (0.65,1.60) |

**Table S6.13** Genotypic and allelic effect of ***IFNGR1* : rs11575936 (Val14Met)** in patients with Beta-lactams (BL) allergy

| **Author, Year** | **Ethnicity** | **HWE P-value** | **Case genotype** | | | **Control genotype** | | | **Case MAF** | **Control MAF** | **Odd ratio (95% CI)** | | | | | |
| --- | --- | --- | --- | --- | --- | --- | --- | --- | --- | --- | --- | --- | --- | --- | --- | --- |
|  |  |  | **GG** | **GA** | **AA** | **GG** | **GA** | **AA** |  |  | **A vs G** | **AA vs GG** | **GA vs GG** | **AA vs GG+GA** | **AA+GA vs GG** | **AA+GG vs GA** |
| Gouglemi et al., 2006 | French | 0.7857 | 21 | 15 | 8 | 13 | 21 | 10 | 0.35 | 0.46 | 0.62  (0.34,1.14) | 0.50  (0.16,1.58) | 0.44  (0.17,1.15) | 0.76  (0.27, 2.14) | 0.46  (0.19,1.10) | 1.77  (0.75,4.17) |
| Gao et al., 2008 | Chinese | 0.8273 | 144 | 0 | 0 | 84 | 4 | 0 | 0.00 | 2.27 | 0.07  (0.00,1.24) | 0.58  (0.01,29.74) | 0.06  (0.00,1.22) | 0.61  (0.01, 31.14) | 0.06  (0.00,1.22) | 15.39  (0.82,289.40) |
| Pooled | | | | | | | | | | | 0.33  (0.05,2.39) | 0.50  (0.17,1.52) | 0.29  (0.06,1.38) | 0.75  (0.27, 2.04) | 0.29  (0.06,1.47) | 3.25  (0.48,21.91) |

**Table S6.14** Genotypic and allelic effect of ***IFNGR2*: rs9808753 (Gln64Arg)** in patients with Beta-lactams (BL) allergy

| **Author, Year** | **Ethnicity** | **HWE P-value** | **Case genotype** | | | **Control genotype** | | | **Case MAF** | **Control MAF** | **Odd ratio (95% CI)** | | | | | |
| --- | --- | --- | --- | --- | --- | --- | --- | --- | --- | --- | --- | --- | --- | --- | --- | --- |
|  |  |  | **AA** | **AG** | **GG** | **AA** | **AG** | **GG** |  |  | **G vs A** | **GG vs AA** | **GA vs AA** | **GG vs AA+AG** | **GG+AG vs AA** | **GG+AA vs AG** |
| Gouglemi et al., 2006 | French | 0.3433 | 36 | 7 | 1 | 33 | 11 | 0 | 0.10 | 0.13 | 0.80  (0.31,2.03) | 2.75  (0.11,69.94) | 0.58  (0.20,1.68) | 3.07  (0.12,77.41) | 0.67  (0.24,1.86) | 1.76  (0.61,5.07) |
| Li et al., 2015 | Chinese | 0.7956 | 20 | 28 | 15 | 12 | 18 | 8 | 0.46 | 0.45 | 1.05  (0.59,1.87) | 1.12  (0.37,3.44) | 0.93  (0.37,2.36) | 1.17  (0.44,3.10) | 0.99  (0.42, 2.36) | 1.12  (0.50, 2.52) |
| Pooled | | | | | | | | | | | 0.98  (0.60,1.59) | 1.26  (0.44,3.57) | 0.76  (0.38,1.52) | 1.29  (0.51, 3.24) | 0.84  (0.43, 1.63) | 1.33  (0.70,2.52) |

**Table S6.15** Genotypic and allelic effect of ***LACTB*: rs2652820** in patients with Beta-lactams (BL) allergy

| **Author, Year** | **Ethnicity** | **HWE P-value** | **Case genotype** | | | **Control genotype** | | | **Case MAF** | **Control MAF** | **Odd ratio (95% CI)** | | | | | |
| --- | --- | --- | --- | --- | --- | --- | --- | --- | --- | --- | --- | --- | --- | --- | --- | --- |
|  |  |  | **GG** | **GA** | **AA** | **GG** | **GA** | **AA** |  |  | **A vs G** | **AA vs GG** | **GA vs GG** | **AA vs GG+GA** | **AA+GA vs GG** | **AA+GG vs GA** |
| Apter et al., 2008 | USA | 0.0824 | 11 | 10 | 2 | 23 | 23 | 1 | 0.30 | 0.27 | 1.21  (0.56,2.63) | 4.18  (0.34,51.24) | 0.91  (0.32,2.55) | 4.38  (0.38,51.04) | 1.05  (0.39,2.84) | 1.25  (0.46,3.40) |
| Cornejo-Garcia et al., 2012 | Spanish | 0.8427 | 28 | 126 | 186 | 27 | 135 | 178 | 0.73 | 0.72 | 1.05  (0.83,1.34) | 1.01  (0.57,1.78) | 0.90  (0.50,1.61) | 1.10  (0.81,1.49) | 0.96  (0.56,1.67) | 1.12  (0.82,1.52) |
| Pooled | | | | | | | | | | | 1.07  (0.85,1.34) | 1.19  (0.63,1.88) | 0.90  (0.54,1.50) | 1.12  (0.83,1.51) | 0.98  (0.61,1.59) | 1.13  (0.84,1.52) |

**Table S6.16** Genotypic and allelic effect of ***TNFa* : rs1800629 (-308G>A)** in patients with Beta-lactams (BL) allergy

| **Author, Year** | **Ethnicity** | **HWE P-value** | **Case genotype** | | | **Control genotype** | | | **Case MAF** | **Control MAF** | **Odd ratio (95% CI)** | | | | | |
| --- | --- | --- | --- | --- | --- | --- | --- | --- | --- | --- | --- | --- | --- | --- | --- | --- |
|  |  |  | **GG** | **GA** | **AA** | **GG** | **GA** | **AA** |  |  | **A vs G** | **AA vs GG** | **GA vs GG** | **AA vs GG+GA** | **AA+GA vs GG** | **AA+GG vs GA** |
| Gueant-Rodriguez et al., 2008 | Italian | 0.0404 | 150 | 15 | 4 | 206 | 47 | 7 | 0.07 | 0.12 | NA | NA | NA | NA | NA | NA |
| Cornejo-Garcia et al., 2012 | Spanish | 0.3756 | 270 | 65 | 5 | 269 | 65 | 6 | 0.11 | 0.11 | 0.97  (0.68,1.38) | 0.83  (0.20,3.31) | 0.99  (0.67,1,48) | 0.83  (1.99,3.30) | 0.98  (0.67,1,45) | 1.00  (0.67,1.49) |
| Pooled | | | | | | | | | | | NA | NA | NA | NA | NA | NA |

**Table S7** subgroup analysis of *IL4R* : rs1801275 (Q576R) by ethnic including predictive interval and false positive reporting probability

| **Gene (Variant)** | **Genetic model** | **OR** | **(95%CI)** | **Predictive interval†** | *** FPRP for prior probability at** | | | | |
| --- | --- | --- | --- | --- | --- | --- | --- | --- | --- |
|  |  |  |  |  | **0.25** | **0.1** | **0.01** | **0.001** | **0.0001** |
| **Non-Asian** | G vs. A | 0.74 | 0.51,1.08 | 0.03, 20.14 | 0.484 | 0.738 | 0.969 | 0.997 | 1.000 |
|  | GG vs. AA | 0.58 | 0.25,1.34 | 0.00,160.88 | 0.555 | 0.789 | 0.976 | 0.998 | 1.000 |
|  | GA vs. AA | 0.77 | 0.31,1.88 | 0.00,2.5e+04 | 0.774 | 0.911 | 0.991 | 0.999 | 1.000 |
|  | GG vs. AA+AG | 0.77 | 0.35,1.71 | 0.00,170.56 | 0.755 | 0.903 | 0.990 | 0.999 | 1.000 |
|  | GG+AG vs. AA | 0.75 | 0.38,1.47 | 0.00,1319.99 | 0.712 | 0.881 | 0.988 | 0.999 | 1.000 |
|  | GG+AA vs. AG | 1.25 | 0.52,3.03 | 0.00, 3.8e+04 | 0.788 | 0.739 | 0.992 | 0.999 | 1.000 |
| **Asian** | G vs. A | 0.74 | 0.50,1.11 | 0.16,3.37 | 0.447 | 0.708 | 0.964 | 0.996 | 1.000 |
|  | GG vs. AA | 0.61 | 0.29,1.28 | 0.10,3.18**^‡^** | 0.547 | 0.784 | 0.976 | 0.998 | 1.000 |
|  | GA vs. AA | 0.69 | 0.43,1.13 | 0.11,4.47**^‡^** | 0.428 | 0.692 | 0.961 | 0.996 | 1.000 |
|  | GG vs. AA+AG | 0.69 | 0.33,1.45 | 0.11,3.62**^‡^** | 0.660 | 0.853 | 0.985 | 0.998 | 1.000 |
|  | GG+AG vs. AA | 0.70 | 0.44,1.12 | 0.12, 4.20 | 0.462 | 0.720 | 0.966 | 0.997 | 1.000 |
|  | GG+AA vs. AG | 1.44 | 0.89,2.32 | 0.23,8.84 | 0.446 | 0.707 | 0.964 | 0.996 | 1.000 |
| **Overall** | G vs. A | **0.73** | **0.57,0.93** | 0.40,1.31 | 0.066 | 0.174 | 0.699 | 0.959 | 0.996 |
|  | GG vs. AA | 0.59 | 0.34,1.04 | 0.27,1.24**^‡^** | 0.275 | 0.532 | 0.926 | 0.992 | 0.999 |
|  | GA vs. AA | 0.71 | 0.49,1.06 | 0.23,2.20**^‡^** | 0.291 | 0.551 | 0.931 | 0.993 | 0.999 |
|  | GG vs. AA+AG | 0.73 | 0.43,1.25 | 0.34,1.52**^‡^** | 0.595 | 0.815 | 0.980 | 0.998 | 1.000 |
|  | GG+AG vs. AA | **0.70** | **0.50,0.99** | 0.28, 1.76 | 0.180 | 0.397 | 0.879 | 0.987 | 0.999 |
|  | GG+AA vs. AG | 1.38 | 0.93,2.05 | 0.45,4.26 | 0.399 | 0.666 | 0.956 | 0.995 | 1.000 |

*FPRP = False positive reporting probability, were reported based on FPRP at noteworthiness 0.2 and were highlighted in grey; †NE= Not estimable due to small sample size; OR = Odds ratio, significant results were reported in bold; **‡**Predictive interval was estimated from random effect model; CI = confident interval; G = Guanine, A = Adenine

**Table S8** subgroup analysis of *IL13* : rs20541, G>A by ethnic including predictive interval and false positive reporting probability

| **Gene (Variant)** | **Genetic model**  **(Minor vs. Major)** | **OR** | **(95%CI)** | **Predictive interval†** | *** FPRP for prior probability at** | | | | |
| --- | --- | --- | --- | --- | --- | --- | --- | --- | --- |
|  |  |  |  |  | **0.25** | **0.1** | **0.01** | **0.001** | **0.0001** |
| **Non-Asian** | A vs. G | 1.17 | 0.98,1.40 | 0.34,4.67 | 0.341 | 0.609 | 0.945 | 0.994 | 0.999 |
|  | AA vs GG | 1.08 | 0.41,2.87 | 0.00, 1.7e+04 | 0.840 | 0.940 | 0.994 | 0.999 | 1.000 |
|  | GA vs GG | **1.41** | **1.07,1.86** | 0.16,12.50 | 0.083 | 0.213 | 0.749 | 0.968 | 0.997 |
|  | AA vs GG+GA | 0.96 | 0.33,2.86 | 0.00, 8.2e+04 | 0.850 | 0.944 | 0.995 | 0.999 | 1.000 |
|  | AA+GA vs GG | 1.37 | 1.08,1.73 | 0.29,6.36 | 0.047 | 0.128 | 0.618 | 0.942 | 0.994 |
|  | AA+GG vs GA | **0.71** | **0.51,0.99** | 0.03,16.20 | 0.197 | 0.424 | 0.890 | 0.988 | 0.999 |
| **Asian** | A vs. G | 0.89 | 0.59,1.32 | NE | 0.780 | 0.914 | 0.992 | 0.999 | 1.000 |
|  | AA vs GG | 0.59 | 0.23,1.53 | NE | 0.623 | 0.832 | 0.982 | 0.998 | 1.000 |
|  | GA vs GG | 1.08 | 0.62,1.89 | NE | 0.825 | 0.934 | 0.994 | 0.999 | 1.000 |
|  | AA vs GG+GA | 0.57 | 0.23,1.40 | NE | 0.578 | 0.804 | 0.978 | 0.998 | 1.000 |
|  | AA+GA vs GG | 0.98 | 0.58,1.66 | NE | 0.850 | 0.944 | 0.995 | 0.999 | 1.000 |
|  | AA+GG vs GA | 0.85 | 0.50,1.44 | NE | 0.773 | 0.911 | 0.991 | 0.999 | 1.000 |
| **Overall** | A vs. G | 1.17 | 0.98,1.04 | 0.78,1.74 | 0.051 | 0.139 | 0.640 | 0.947 | 0.994 |
|  | AA vs GG | 0.90 | 0.43,1.86 | 0.07,11.21 | 0.824 | 0.934 | 0.994 | 0.999 | 1.000 |
|  | GA vs GG | **1.34** | **1.07,1.68** | 0.81,2.22 | 0.063 | 0.168 | 0.689 | 0.957 | 0.996 |
|  | AA vs GG+GA | 0.82 | 0.38,1.76 | 0.05,13.34 | 0.786 | 0.917 | 0.992 | 0.999 | 1.000 |
|  | AA+GA vs GG | **1.29** | **1.04,1.61** | 0.80,2.08 | 0.127 | 0.304 | 0.828 | 0.980 | 0.998 |
|  | AA+GG vs GA | **0.74** | **0.59,0.92** | 0.37,1.45 | 0.50 | 0.137 | 0.636 | 0.946 | 0.994 |

*FPRP = False positive reporting probability, were reported based on FPRP at noteworthiness 0.2 and were highlighted in grey; †NE= Not estimable due to small sample size or contain one study; OR = Odds ratio, significant results were reported in bold; **‡**Predictive interval was estimated from random effect model; CI = confident interval; G = Guanine, A = Adenine

**Table S9** Sensitivity analysis of *IL4R*: rs1801275 (Q576R) when a given name study is omitted

| **Author, Year** | **Ethnicity** | **Estimated OR** | **95% CI** |
| --- | --- | --- | --- |
| Qiao et al., 2005 | Chinese | 0.77 | 0.65, 0.91 |
| Gouglemi et al., 2006 | Caucasian | 0.72 | 0.61,0.85 |
| Gueant-Rodriguez et al., 2006 | Italian | 0.77 | 0.64,0.92 |
| Huang et al., 2009 | Chinese | 0.79 | 0.65,0.95 |
| Zhang et al., 2010 | Chinese | 0.72 | 0.61,0.85 |
| Li et al., 2015 | Chinese | 0.72 | 0.61,0.84 |
| Khadhim et al., 2018 | Iraq | 0.73 | 0.62,0.87 |

**Table S10** Sensitivity analysis of *IL13*: rs20541, G>A when a given name study is omitted

| **Author, Year** | **Ethnicity** | **Estimated OR** | **95% CI** |
| --- | --- | --- | --- |
| Yang et al., 2005 | Chinese | 1.26 | 1.06,1.49 |
| Gouglemi et al., 2006 | French | 1.20 | 1.03,1.40 |
| Gueant-Rodriquez et al., 2006 | Italian | 1.11 | 0.93,1.33 |
| Cornejo-Garcia et al., 2012 | Spanish | 1.27 | 1.06,1.52 |
